# Food-induced anaphylaxis to plant allergens in adolescents and adults: Data from the allergy Vigilance Network (2002–2024)^☆^

**DOI:** 10.1016/j.waojou.2026.101448

**Published:** 2026-08-13

**Authors:** Naphisabet Wanniang, Françoise Codreanu-Morel, Guillaume Pouessel, Isabela Assugeni, Amandine Divaret-Chauveau, Dominique Sabouraud-Leclerc, Annette Kuehn

**Affiliations:** a Department of Infection and Immunity, Luxembourg Institute of Health, Esch-sur-Alzette, Luxembourg; b Faculty of Science, Technology and Medicine, University of Luxembourg, Esch-sur-Alzette, Luxembourg; c Department of Allergology and Immunology, Centre Hospitalier de Luxembourg-Kanner Klinik, Luxembourg; d Allergy-Vigilance Network, Vandœuvre-lès-Nancy, France; e CH Roubaix, Department of Paediatrics, Children's Hospital, Roubaix, France; f CHU Lille, Paediatric Pulmonology and Allergy Department, Pole Enfant, Hôpital Jeanne de Flandre, Lille, France; g Univ Lille, ULR 2694: METRICS, Lille, France; h Paediatric Allergy Unit, Children's Hospital, University Hospital of Nancy, Vandoeuvre les Nancy, France; i UR 3450 DevAH, Health Faculty, Vandoeuvre les Nancy, France; j General and Specialised Paediatrics, Department, American Hospital, Reims University Hospital, Reims, France

**Keywords:** Anaphylaxis, Plant-based diet, Food allergy, Food-induced anaphylaxis

## Abstract

**Background:**

There is a growing interest in plant-based diets (PBDs) owing to their health benefits and environmental sustainability. However, plant proteins commonly consumed in PBDs are recognized allergens and are known triggers of food-induced anaphylaxis (FIA).

**Aim:**

To characterize the clinical phenotypes of FIA due to plant allergens relevant to PBDs (“PAs”) reported to the Allergy-Vigilance Network in adolescents and adults.

**Methods:**

Anaphylaxis cases attributed to plant allergens in individuals ≥13 years of age were retrospectively analyzed (2002–2024). Cases were identified by culprit allergen, irrespective of dietary patterns, and assessed for time trends, eliciting dose, cofactors, and component-resolved diagnostics. Age-specific phenotypes were compared between adolescents and adults.

**Results:**

753 plant allergen-related FIA cases were recorded, 237 (31.5%) occurred in adolescents and 516 (68.5%) in adults. Eleven allergens (wheat, peanut, buckwheat, soy, hazelnut, sesame, lupin, cashew, pine nut, walnut and almond) accounted for 85% of all PBD-related FIA. Wheat was the main elicitor in adults (26.9% adults vs 7.6% adolescents; *P* < 0.001). Peanut predominated in adolescents (34.2% adolescents vs 8.1% adults; *P* < 0.001), and was associated with pre-existing peanut-allergy (71.8% adolescents vs 46.2% adults; *P* = 0.012). Soy cases in adults were frequently associated with birch pollen allergy (18.8% adolescents vs 64.5% adults; *P* = 0.005), whereas soy-related FIA cases in adolescents occurred in the context of a prior legume-allergy diagnosis (50% adolescents vs 5.6% adults; *P* < 0.001). In adolescents, physical exercise was the main cofactor reported, whereas adults showed a wider range including physical exercise, alcohol, and medications.

**Conclusion:**

Several plant allergens displayed distinct age-specific clinical phenotypes, which may be relevant for dietary counseling in food-allergic or atopic individuals considering adoption of PBDs.

## Introduction

Plant-based diets (PBDs) represent a diverse spectrum of dietary patterns prioritizing plant-derived foods with the optional inclusion of small amounts of animal products. PBDs are highly encouraged for human health and environmental sustainability.^1^^,^^2^ The EAT-Lancet Commission advocates PBDs for their potential health benefits, including reduced risk of non-communicable diseases.^3^^,^^4^ The European Food Safety Authority (EFSA) provides further scientific guidance to this.^5^ PBDs encompass various dietary approaches such as vegetarian, and flexitarian diets (Supplemental Table 1).^2^ A vegan diet, by contrast, strictly excludes all foods wholly or partially derived from animals.^6^ An increasing proportion of the Western population identifies as flexitarian, meaning individuals who predominantly follow a PBD while occasionally consuming meat and animal-derived products.^7^^,^^8^ A 2023 survey across Europe reported that 1–5% of the adult population identified as vegan, 4–6% as vegetarians and 16–40% as flexitarian. Additionally, 53% of European adults plan to increase their legumes consumption while 40% intend to opt for more plant-based alternatives.^7^ In parallel with changing consumer dietary habits, the European market has responded with a marked increase in the availability of plant-based food products. Based on new product launches tracked in a commercial food database and reported by an industry association, launches containing plant-based proteins increased between 2012 and 2017 (average annual growth, 13.5%).^9^ Although wheat and soy dominated the launches in 2017 (84%), the market progressively diversified with pea protein emerging as a significant contributor, increasing from 0.9% to 12% of launches between 2007 and 2017 respectively.^9^ This growth has continued, with global alternative-protein product launches rising at a 10% annual rate between 2020 and 2024.^10^

This transition may have important implications for immunoglobulin E (IgE)-mediated food allergy (FA), the most common immunological form, which carries a risk of anaphylaxis.^11^ Whether exposure to novel or increasingly consumed plant-derived proteins is associated with changes in allergic sensitization and clinical allergy profiles remains unknown.^12^, ^13^, ^14^ The most common elicitors of food-induced anaphylaxis (FIA) in children include peanut, tree nuts (cashew, hazelnut), cow's milk, and hen's egg, whereas wheat, shellfish, peanut, hazelnut, and soy are frequently implicated in adults.^15^, ^16^, ^17^, ^18^, ^19^, ^20^, ^21^ Despite the growing adoption of PBDs, data on their coexistence with FA remain limited. This represents a clinically relevant gap.^22^ PBDs rely on a group of protein-rich plants, including legumes, cereals, nuts and seeds to meet the recommended protein intake, many of which are recognized food allergens.^23^, ^24^, ^25^, ^26^ Individuals with allergies to plant proteins may face substantial challenges when attempting to reduce or eliminate animal-derived products.^27^ Moreover, there is a lack of clear, evidence-based dietary guidance for food-allergic individuals seeking to adopt more PBDs. Overall, the relationship between PBDs and FA remains complex and is challenging to comprehensively address due to emerging allergens, cross-reactivities, and limited population-level data. A pragmatic approach is to focus on individuals with established FA, as identified through food anaphylaxis registries. Such registries record reactions by culprit allergen, irrespective of dietary patterns; cases therefore reflect real-world exposure to these allergens regardless of individual diet.

This study aims to characterize the clinical phenotypes of FIA attributed to plant allergens relevant to PBDs in adolescents and adults, using data from a large cohort of FIA cases reported to the Allergy-Vigilance Network (AVN) over a 23-year period (2002–2024). These age groups were included because dietary habits, including PBD adoption, are typically established from adolescence onwards. Characterizing these phenotypes may help guide the counseling of food-allergic or atopic patients who wish to adopt a PBD.

## Methods

### Study design

The AVN is a registry that has been collecting cases of anaphylaxis since 2002, reported by allergists from French-speaking countries (specifically France, Belgium, and Luxembourg). Cases are recorded using a structured online questionnaire comprising more than 70 variables, including demographics data, relevant clinical information, results of allergy workup (skin prick tests, specific IgE and/or component-resolved diagnostics [CRD]). All submitted cases are reviewed and validated by an expert AVN panel prior to inclusion in the database.

The study complies with the European General Data Protection Regulation and the French national data protection framework. It was approved by the National Committee of Data and Patient Protection. All data were collected anonymously in accordance with French data protection regulations, and informed consent was obtained from all participants or their relatives.

### Data extraction, variables and age-group definition

Demographic, clinical variables extracted include age, sex, causative allergen, food type, quantity consumed, cofactors involved, atopic comorbidities, and CRD, when available. Only anaphylaxis cases classified as ≥ grade 2 according to Ring modified by Behrendt classification were included.^28^ Eliciting doses (ED) are expressed in kitchen measurements as described by Faust et al.^29^ Year of declaration was collected for time-trend analyses for FIA cases declared to AVN. Analyses focused on FIA caused by plant proteins relevant to PBDs, including legumes, tree nuts, cereals, seeds and pseudocereals.^23^ These proteins are referred to as “*plant allergens (PAs)*”. Age at reaction was categorized as adolescents (13–19 years) and adults (>19 years), adapted from the World Health Organization definition and Grabenhenrich et al.^30^^,^^31^ Clinical phenotype analyses were limited to the most frequent plant allergen (PA) elicitors of FIA in adolescents and adults. As secondary analysis, the distribution of PA-related FIA was also collected for infants (<1 year) and children (1 to <13 years).

### Statistical analysis

All statistical analyses were performed using R software (version 4.3.1). Qualitative data are presented as counts and percentages. Non-normally distributed quantitative data are expressed as median and interquartile range (IQR). Time trends in annual case counts were assessed using Poisson regression models to estimate the annual percent change (APC) and its 95% confidence intervals (CIs). To test whether the trend was constant over the study period, a segmented Poisson model was fitted with the breakpoint estimated from the data, and a change in slope was evaluated using the Davies test. All descriptive analyses are based on available data without imputation. Missing values were excluded from percentage calculations. Fisher's exact or chi-square test was used to compare categorical variables between groups and the Mann-Whitney *U* test was used to evaluate differences in continuous variables. Clinical and demographic characteristics were compared as separate, pre-specified analyses without adjustment. The Benjamini-Hochberg correction was applied to all per-allergen analyses (age group distribution and APC) and to CRD comparisons involving 3 or more categories. P value < 0.05 was considered statistically significant.

## Results

Among the 3153 Ring grade ≥2 FIA cases reported to the AVN, 2217 (70.3%) involved plant-derived elicitors, all ages combined. Of these, 1694 (76.4%) were due to PAs. Four cases were excluded (age not specified) resulting in 1690 cases. Among these, adolescents (N = 237; 14%) and adults (N = 516; 30.5%) together accounted for 44.5% of cases. Additionally, FIA induced by PAs occurred in 909 (53.8%) cases in children and in 28 (1.7%) cases in infants (Fig. 1).

**Fig. 1:**
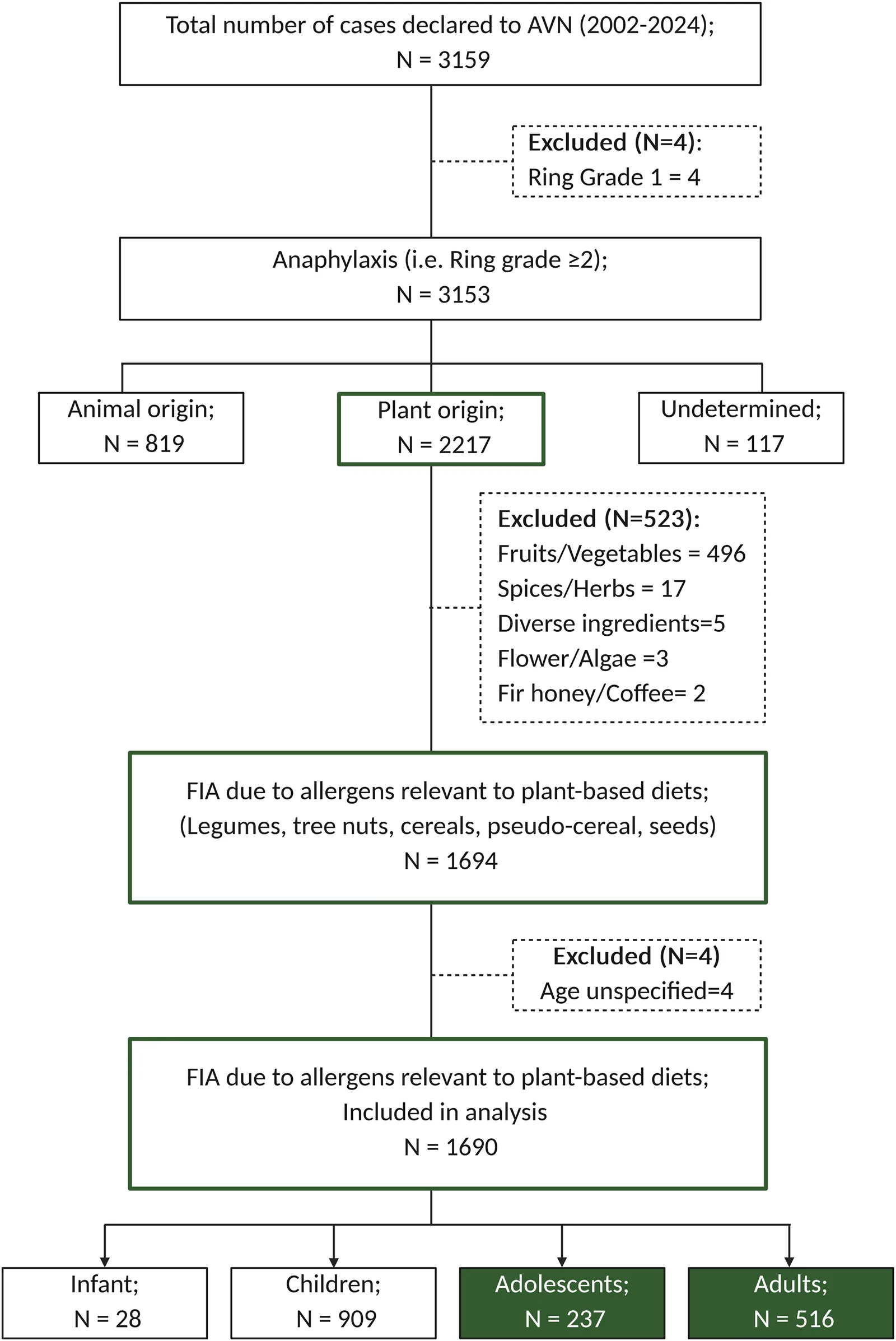
Flowchart of the food-induced anaphylaxis cases collected by the Allergy-Vigilance Network: 2002–2024 and included in our final analysis (highlighted in green); Undetermined category includes idiopathic or unknown allergens, food coloring, and additives/preservatives.

### Time trend of all FIA attributed to plant allergens relevant to PBDs over 2 decades (2002–2024)

Among adolescents, PA-related FIA remained stable until approximately 2017, after which cases declared increased significantly (post-breakpoint APC 16.7%; 95% CI 6.3–28.1; *P* = 0.011). In contrast, cases declared remained stable among adults (−0.8% per year; 95% CI –2.1 to 0.5; *P* = 0.230) (Fig. 2A). At the individual-allergen level, peanut showed a significant increase in adolescents (APC 5.5%; 95% CI 1.9–9.2; N = 79; *P* = 0.020) whereas trends for cashew, pistachio, and walnut were based on few cases (N = 10–14) with wide CIs (Fig. 2B). In adults, no allergen increased significantly, whereas significant decreases were observed for lupin (APC −12.2%; 95% CI −17.3 to −6.9; N = 37; *P* < 0.001) and buckwheat (APC −6.7%; 95% CI −11.0 to −2.1; N = 44; *P* = 0.024) (Fig. 2C). These per-allergen trends were based on small annual case counts and should be interpreted with caution.

**Fig. 2:**
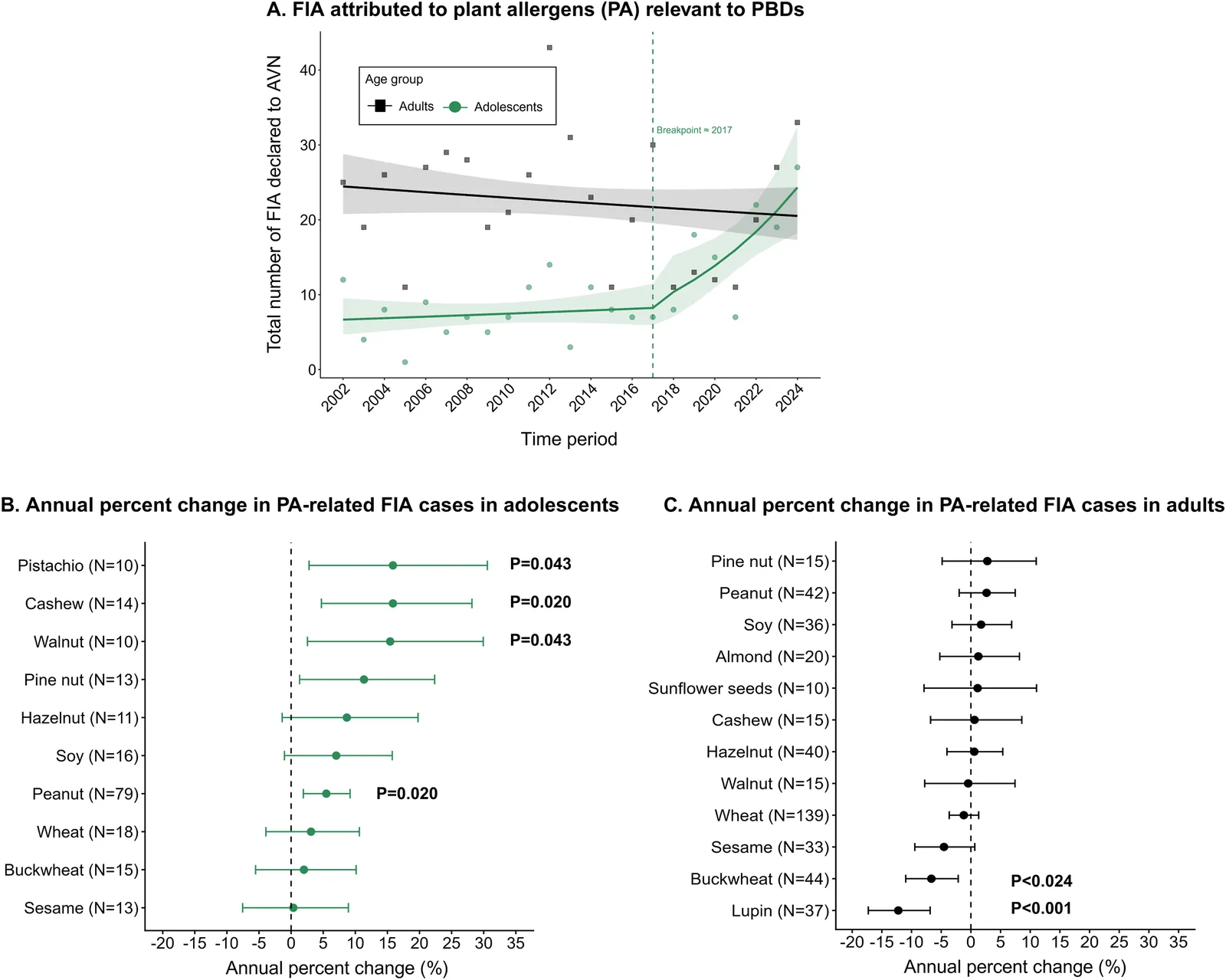
(A) Annual total FIA cases attributed to plant allergens (PAs) reported to the Allergy-Vigilance Network, 2002–2024, by age group. Points are annual counts; lines are fitted Poisson trends with 95% confidence bands. A segmented model was fitted to both age groups; a significant breakpoint was identified only in adolescents (≈2017). For adults, no significant breakpoint was identified, and the trend is shown as a single fitted slope. Annual percent change (APC) in FIA attributed to specific PA among (B) Adolescents and (C) Adults. For clarity, only allergens with ≥10 reported cases over the 2002–2024 study period are displayed. Error bars represent 95% confidence intervals. Trend analyses included cases with a documented year. N represents number of cases. P values (per-allergen APC) were derived from Poisson regression and Benjamini-Hochberg adjusted within each age group

### Age distribution of food-induced anaphylaxis due to plant allergens

Fig. 3 shows the distribution of PA families within each age group. In adolescents, legumes (48.5%) and tree nuts (21.9%) accounted for the majority of PA-related FIAs while in adults, cereals (29.7%), legumes (26.6%), and tree nuts (21.1%) predominated.

**Fig. 3:**
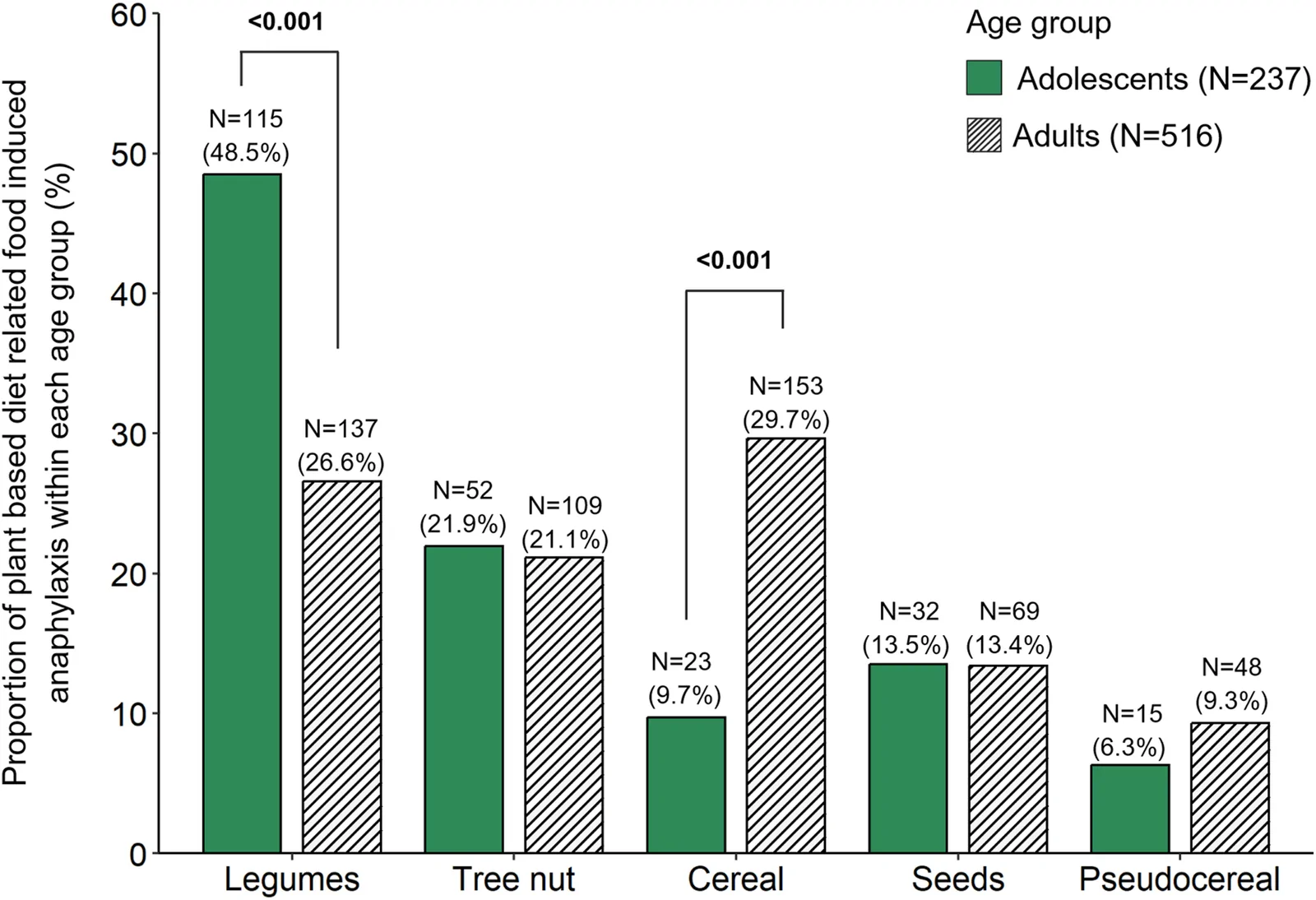
Age distribution of food-induced anaphylaxis cases attributed to plant allergens relevants to plant-based diets reported to the Allergy-Vigilance Network (2002–2024), stratified by age group. P-values shown were calculated using Fisher's exact test, with Benjamini-Hochberg correction for multiple comparisons across allergen families. Only comparisons reaching statistical significance are indicated

Supplemental Fig. 1 illustrates the relative contribution of specific allergens within each PA families to FIA. In adolescents, peanut (70.4%) predominated among legumes, whereas peanut, soy, and lupin contributed similarly in adults (26–30%). Other legumes commonly used in PBDs, such as peas (1.7% in adolescent vs 1.5% in adults) and lentils (2.9% in adults), were uncommon elicitors of FIA in both age groups. Tree nut-related FIA in adolescents was mainly due to cashew (26.9%), hazelnut (21.2%), pistachio (19.2%) and walnut (19.2%), while hazelnut (36.7%) and almond (18.3%) were the leading elicitors in adults. Sesame (40.6% vs 47.8%) and pine nut (40.6% vs 21.7%) were the most common seed allergens in adolescents and adults, respectively. Wheat was the dominant cereal allergen (78.3% vs 90.8%), and buckwheat accounted for nearly all pseudocereal-related FIA (100% vs 91.7%) in adolescents and adults respectively.

### Clinical phenotype and immunological profile of the most frequent plant allergens: comparison according to age group

Fig. 4 depicts the most frequent PAs responsible for FIA in adolescents and adults. Eleven allergens represent 85% (640/753) of PA-related FIA.

**Fig. 4:**
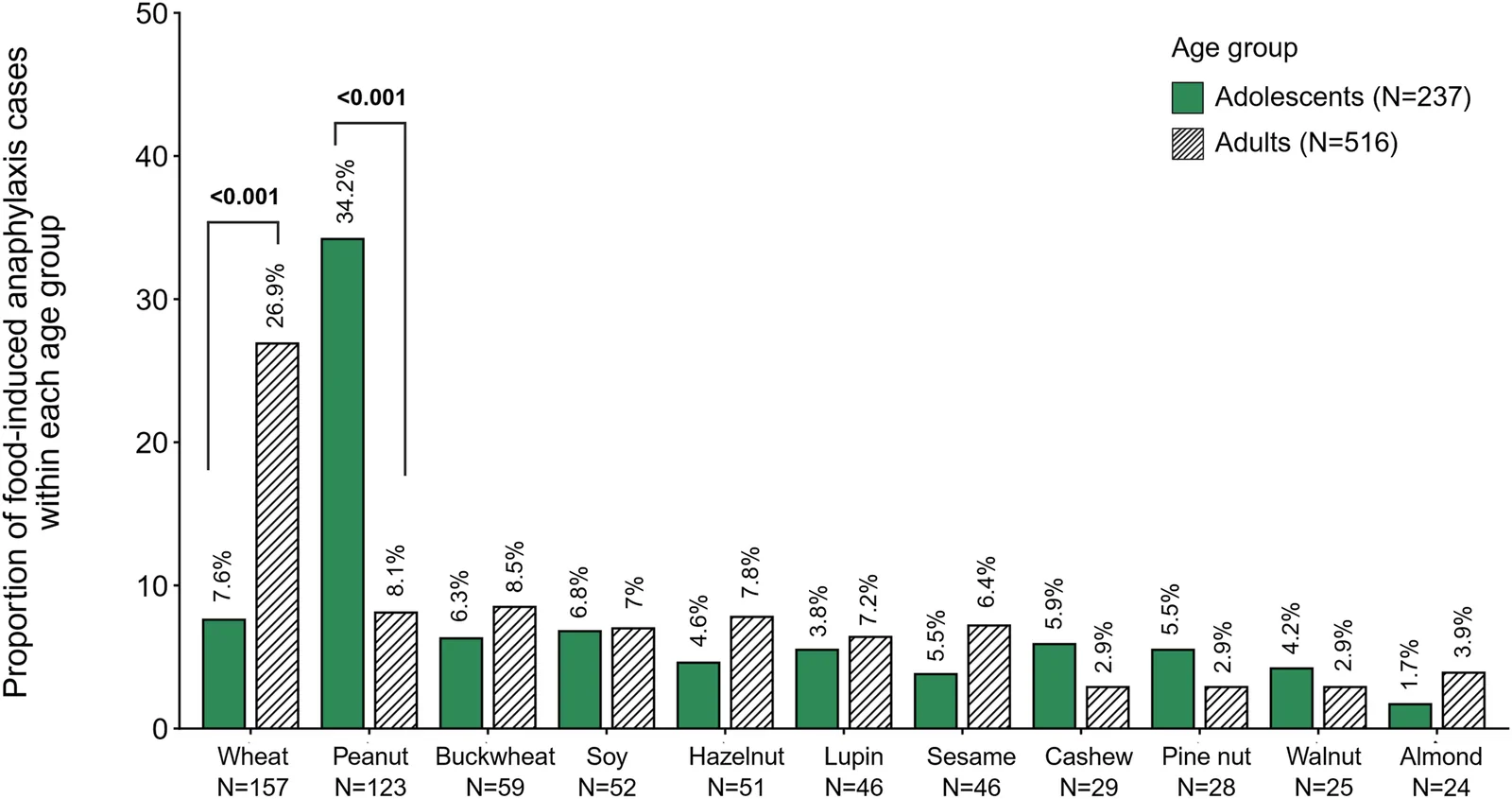
Proportion of food-induced anaphylaxis (FIA) cases attributed to the eleven most frequent plant allergens in adolescents and adults. Percentages are calculated using the total number of FIA cases within each age group for the respective allergen as the denominator. The total number of cases for each allergen across adolescents and adults is shown on the x-axis labels. P-values shown were calculated using Fisher's exact test, with Benjamini-Hochberg correction for multiple comparisons across allergen. Only comparisons reaching statistical significance are indicated

Clinical phenotype of these allergens are presented in descending order of frequency, as shown in Fig. 4. Additionally, Fig. 5 summarizes our findings and observed patterns of FIA due to PAs in adolescents and adults, including age-associated phenotypic presentation and related clinical considerations.

**Fig. 5:**
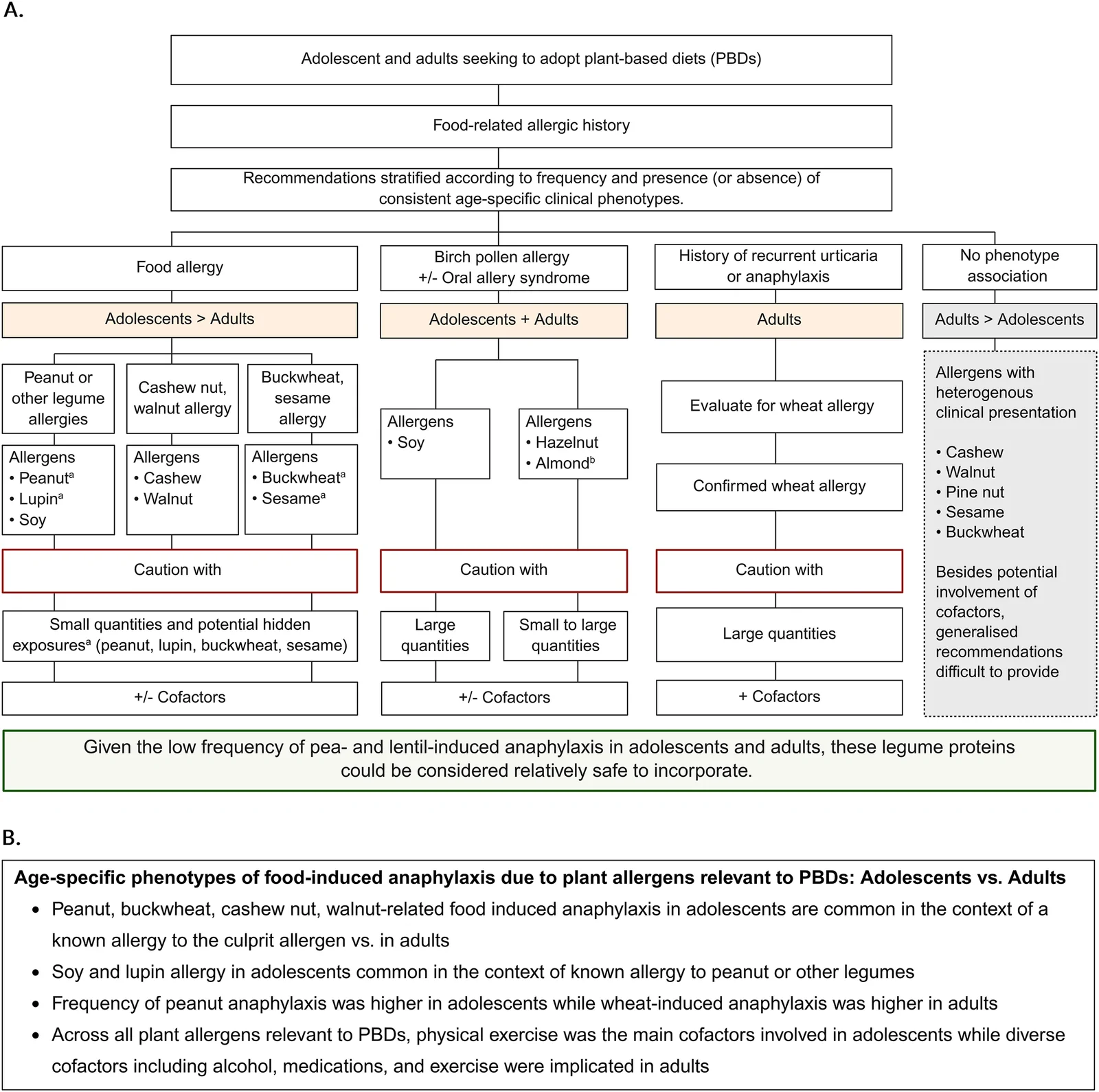
Summary of the study findings, translated into the situation of adolescents/adults seeking to adopt plant-based diets (PBDs). (A) Overview of the observed patterns of food-induced anaphylaxis (FIA) associated with plant allergens relevant to PBDs (PAs) in adolescents and adults and possible clinical considerations. (B) Age-specific phenotypes of FIA associated with the main PAss in adolescents versus adults. ^a^PAs with possible risk of reactions following hidden exposures; ^b^Reflects the adult phenotype; the small number of adolescent cases limits recommendations for this age group

### Wheat

One-hundred fifty-seven cases of wheat-induced anaphylaxis were reported. Most occurred in adults (N = 139; 88.5%) while adolescents accounted for 11.5% (N = 18) of cases (Table 1). Grade 2 reactions occurred in 66.7% of adolescents and 49.6% of adults, grade 3 in 33.3% and 48.2%, respectively, and grade 4 occurred only in adult (2.2%). No fatalities were reported. Cofactors were frequently reported, 94.4% in adolescents and 90.5% in adults (Supplemental Table 2). Physical exercise (PE) was the most common cofactor, reported in 70.6% adolescents and 50% adults. In addition, combinations of cofactors, medications and alcohol, were also observed in adults (Supplemental Fig. 2A). A prior wheat-allergy diagnosis was uncommon in both adolescents (5.9%) and adults (10.9%), whereas self-reported symptoms following wheat consumption, including recurrent urticaria or anaphylaxis, were frequent (64.7% and 60.3%, respectively). Sensitization to Tri a 19 was more frequent in adults (97.7%) vs adolescents (72.7%; *P* = 0.009), whereas Tri a 14 sensitization was more common in adolescents vs adults (27.3% vs 2.3%, *P* = 0.009; Supplemental Table 2). Most reactions (78.9%) occurred after full serving-size meals (Supplemental Table 3), commonly bread or pizza. In most cases (96.6%), wheat was identifiable in the consumed food products while it was not readily recognizable in only 5 cases (soup packets, soy sauce, and meat preparations) (Supplemental Table 4).

**Table 1:** Age distribution of all food-induced anaphylaxis attributed to plant allergens relevant to plant-based diets.

Food-induced anaphylaxis attributed to plant allergens in adolescents and adults (N = 753)			
Allergens	Total number of cases	Adolescents N (%)	Adults N (%)
**Legumes**	252	115 (45.6)	137 (54.7)
**Peanut**	**123**	**81 (65.9)**	**42 (34.1)**
**Soy**	**52**	**16 (30.8)**	**36 (69.2)**
**Lupin**	**46**	**9 (19.6)**	**37 (80.4)**
Fenugreek	6	3 (50)	3 (50)
Mung beans	5	2 (40)	3 (60)
Lentils	4	0 (0)	4 (100)
Peas	4	2 (50)	2 (50)
Beans	4	0 (0)	4 (100)
Chickpeas	2	1 (50)	1 (50)
Mix of legumes	2	0 (0)	2 (100)
Broad beans	1	0 (0)	1 (100)
Carob	1	0 (0)	1 (100)
Alfalfa sprouts	1	1 (100)	0 (0)
Flageolet beans	1	0 (0)	1 (100)
**Cereals**	176	23 (13.1)	153 (86.9)
**Wheat**	**157**	**18 (11.5)**	**139 (88.5)**
Barley	7	0	7 (100)
Maize	7	1 (14.3)	6 (85.7)
Oats	3	2 (66.7)	1 (33.3)
Rice	2	2 (100)	0 (0)
**Tree nuts**	161	52 (32.3)	109 (67.7)
**Hazelnut**	**51**	**11 (21.6)**	**40 (78.4)**
**Cashew**	**29**	**14 (48.3)**	**15 (51.7)**
**Walnut**	**25**	**10 (40)**	**15 (60)**
**Almond**	**24**	**4 (16.7)**	**20 (83.3)**
Pistachio	14	10 (71.4)	4 (28.6)
Brazil nut	8	2 (25)	6 (75)
Mix of tree nuts	6	1 (16.7)	5 (83.3)
Macadamia	3	0 (0)	3 (100)
Pecan	1	0 (0)	1 (100)
**Seeds**	101	32 (31.7)	69 (68.3)
**Sesame**	**46**	**13 (28.3)**	**33 (71.7)**
**Pine nut**	**28**	**13 (46.4)**	**15 (53.6)**
Sunflower seeds	11	1 (9.1)	10 (90.9)
Mustard	7	3 (42.9)	4 (57.1)
Flax seed	4	0 (0)	4 (100)
Pumpkin seed	4	2 (50)	2 (50)
Chia seeds	1	0 (0)	1 (100)
**Pseudocereal**	63	15 (23.8)	48 (76.2)
**Buckwheat**	**59**	**15 (25.4)**	**44 (74.6)**
Quinoa	3	0 (0)	3 (100)
Amaranth	1	0 (0)	1 (100)

Allergens are presented in descending order according to the frequency of reported cases, first by plant allergen families and then of individual allergen within each family

### Peanut

Of 123 peanut-induced anaphylaxis cases, 81 (65.9%) occurred in adolescents and 42 (34.1%) in adults. (Table 1). Most reactions were grade 2–3 in both age groups, with grade 4 occurring in 9.9% of adolescents vs 4.8% of adults (*P* = 0.641) including 4 fatalities in adolescents vs 1 in adult. Cofactors were reported more frequently in adults (50%) than in adolescents (25.3%; *P* = 0.016; Supplemental Table 5), with alcohol being the main trigger in adults (47.4%), whereas diverse cofactors were reported in adolescents (Supplemental Fig. 3A). A higher proportion of adolescents had a previously diagnosed peanut-allergy compared with adults (71.8% vs 46.2% respectively; *P* = 0.012; Supplemental Table 5). Most reactions followed small exposures (<1 teaspoon; 54.9%), mainly from peanut-containing dessert or snacks (Supplemental Table 3). In 22.3% of cases, peanut was not identifiable in the consumed product due to contamination or labeling issues (Supplemental Table 4).

### Buckwheat

Among 59 buckwheat-induced anaphylaxis cases reported, most occurred in adults (N = 44; 74.6%) vs adolescents (N = 15; 25.4%; Table 1). Reactions were predominantly grade 2 (73.3% in adolescents vs 61.4% in adults) with grade 3 occurring in 26.7% vs 38.6% respectively. No grade 4 reactions were observed. Cofactors were reported in 26.7% of adolescent and 35.7% of adult cases (Supplemental Table 6). PE was the most common cofactor in adolescents (50%), whereas adults more frequently reported multiple cofactors, alcohol, and medications (Supplemental Fig. 2B). Previous buckwheat-allergy diagnosis was more frequent in adolescents (46.7%) vs adults (13.9%; *P* = 0.026; Supplemental Table 6). Most reactions (59%) followed ingestion of a full-serving portions (eg, buckwheat crepes, bread, or pastries), while 23.1% occurred after very small exposures (<1 teaspoon, Supplemental Table 3). Reactions attributed to hidden exposure due to either contamination or lack of labelling occurred in 9 cases (16.7%; Supplemental Table 4).

### Soy

Fifty-two cases of soy-induced anaphylaxis were reported, mainly in adults (N = 36; 69.2%) vs adolescents (N = 16; 30.8%; Table 1). The majority of reactions were grade 2 (81.2% in adolescents and 80.6% in adults), while grade 3 reactions accounted for 18.8% and 19.4% of cases, respectively, with no fatalities reported. Cofactors were reported with similar frequency in adolescents (37.5%) and adults (44.4%; Supplemental Table 5). PE was the most frequent cofactor in adolescents (50%). In adults, PE was relatively less common (25%), while medications and combination of cofactors (∼60%) predominated (Supplemental Fig. 3B). Prior soy-allergy diagnosis was uncommon in both groups (∼7–13%). Adolescents more frequently reported a known peanut and/or other legume-allergy diagnosis (50%) compared to adults (5.6%; *P* < 0.001). Sensitization to seed storage proteins (Gly m 5/6) was also more frequent in adolescents (45.5%) compared to adults (4%; *P* = 0.018). In contrast, birch pollen allergy (BPA) was more frequently reported in adults than in adolescents (64.5% vs 18.8%; *P* = 0.005) and were associated with a sensitization to Gly m 4, a pathogenesis-related protein 10 (PR-10) (84% vs 45.5%; *P* = 0.059; Supplemental Table 5). Soy beverages were the most frequently implicated products in adults (70.6%), whereas a broader range of food products was reported in adolescents, including soy beverages (37.5%), meat preparations (18.8%), meat alternatives (12.5%), and ultra-processed soy products (12.5%). FIA induced by soy beverages were more frequently reported in patients sensitized exclusively to Gly m 4 (50%), while those sensitized to Gly m 5 and/or Gly m 6 reported a broader range of food sources (Supplemental Table 7). Majority of reactions (60.7%) occurred after ingestion of large amount (1/2 cup–1 plate; Supplemental Table 3). Soy was identifiable (95.7%), with contamination or mislabeling reported in only 2 cases (Supplemental Table 4).

### Hazelnut

Fifty-one hazelnut-induced anaphylaxis cases were reported, 78.4% (N = 40) in adults and 21.6% (N = 11) in adolescents (Table 1). Reaction severity was similar between groups, with grade 2 predominating (63.6% in adolescents vs 65% in adults), grade 3 occurring in 36.4% and 30%, respectively, and grade 4 observed only in adults (5%) including 1 fatality. Cofactors were reported at similar frequencies in adolescents (54.5%) and adults (63.2%; *P* = 0.729; Supplemental Table 8). PE (33.3%) and peak pollen season (33.3%) were the main cofactors in adolescents, whereas alcohol (45.8%) predominated in adults (Supplemental Fig. 4A). Prior hazelnut-allergy diagnosis was more frequent in adolescents vs adults (40% vs 14.7%; *P* = 0.175), as were BPA (54.5% vs 34.3%; *P* = 0.296), and oral allergy syndrome (OAS) (50% vs 20.5%; *P* = 0.104). Among the cases with available CRD data — a minority of patients — sensitization to Cor a 1 and to Bet v 1 (both PR-10) were common in both age groups. Sensitization to seed storage proteins (Cor a 9/14) was observed in 2 adolescents (28.6%; Supplemental Table 8). Most reactions occurred after small exposures (<1 teaspoon-1 tablespoon; 70.6%), typically via hazelnut-containing desserts, snacks, or native hazelnut (Supplemental Table 3). Hazelnut was identifiable in most cases with unrecognized exposure occurring in only 3 cases (1 chocolate and 2 home-made cakes without labeling; Supplemental Table 4).

### Lupin

Among 46 cases of lupin-induced anaphylaxis, most occurred in adults (N = 37; 80.4%), with a female predominance (81.1%), while 19.6% (N = 9) occurred in adolescents. Reactions were mainly grade 2 in both groups (100% in adolescents vs 81.1% in adults). Grade 3 reactions occurred in 18.9% of adults, with no fatalities reported. Cofactors were infrequent (12.5% in adolescents vs 20.6% in adults; Supplemental Table 5). Previous lupin-allergy diagnosis was reported in 28.6% of adolescents and 33.3% of adults and prior peanut or other legume-allergy diagnosis was more common in adolescents than in adults (37.5% vs 9.4% respectively, *P* = 0.082). Most reactions (66.7%) followed consumption of a full serving of lupin flour-containing pastries, with only 1 case reported after ingestion of a small amount (<1 teaspoon; Supplemental Table 3). In 19% of the cases, reactions were suspected to be induced by lupin in industrial food products although it was not clearly identifiable (Supplemental Table 4).

### Sesame

Of the 46 sesame-induced anaphylaxis cases, 71.7% (N = 33) occurred in adults and 28.3% (N = 13) in adolescents (Table 1). Reactions were predominantly grade 2 (69.2% in adolescents vs 54.5% in adults), with grade 3 observed in 30.8% vs 45.5% respectively. No grade 4 or fatalities occurred. Cofactors were reported at similar frequencies in adolescents (38.5%) and adults (35.5%; Supplemental Table 9), with PE being common in adolescents (40%), whereas multiple cofactors (36.4%) and alcohol (27.3%) predominated in adults (Supplemental Fig. 5A). Previous sesame-allergy diagnosis was more frequent in adolescents (36.4%) than in adults (10.7%; *P* = 0.083). BPA was reported in 27.3% of adolescents and 3.7% of adults (*P* = 0.065; Supplemental Table 9). Most reactions (60%) followed exposure to small amounts (<1 teaspoon to 1 tablespoon), typically via sesame paste (tahini or hummus), whereas 40% occurred after consumption of full serving-size sesame-containing meals (eg, burgers, biscuits; Supplemental Table 3). Reactions due to hidden exposure was seen in 14.6% of cases, all attributed to mislabeling (Supplemental Table 4).

### Cashew

Of the 29 cashew–induced anaphylaxis cases, 48.3% (N = 14) occurred in adolescents while 51.7% (N = 15) in adults (Table 1). The majority of reactions were grade 2 (78.6% in adolescents vs 53.3% in adults), while grade 3 reactions occurred in 21.4% and 46.7%, respectively. No grade 4 reactions or fatalities were reported. Cofactors were reported only in adults (38.5%) and none in adolescents (*P* = 0.015; Supplemental Table 8) with alcohol (60%) and medications (40%) being the main cofactors (Supplemental Fig. 4C). Previous cashew-allergy diagnosis was more frequent in adolescents than in adults (50% vs 0%; *P* = 0.006) (Supplemental Table 8). Majority of reactions (81.3%) followed ingestion of small amounts (<1 teaspoon-1 tablespoon; Supplemental Table 3), typically cashew in its native form or cashew-based sauces. Cashew was identifiable in nearly all cases, with only 1 reaction strongly suspected to be due to cashew despite it not being identifiable in a sauce. (Supplemental Table 4).

### Pine nut

Twenty-eight pine nut–induced anaphylaxis cases were reported which were similarly distributed between adolescents (46.4%; N = 13) and adults (53.6%; N = 15; Table 1). Reactions were predominantly grade 2 (84.6% in adolescents vs 60% in adults), while grade 3 reactions occurred in 15.4% and 40%, respectively, with no grade 4 reactions observed. Cofactors were uncommon in adolescents, reported in only 1 case (7.7%), attributed to stress, whereas cofactors were present in 50% of adult cases (*P* = 0.030; Supplemental Table 9), mostly alcohol (33.3%; Supplemental Fig. 5B). Prior pine nut-allergy diagnosis was uncommon in both age groups. BPA was observed only in adolescents (25%), while OAS was reported in 8.3% of adolescents and 21.4% of adults (*P* = 0.598; Supplemental Table 9). Most reactions (78.6%) followed small exposures (<1 teaspoon–1 tablespoon), typically from native pine nuts in salads or pesto, while 3 cases (21.4%) occurred after consumption of full serving-size of pine nut-based meals (eg, sandwich, tartare, dessert; Supplemental Table 3). Pine nut was not identifiable in 19.2% of cases due to either contamination or lack of labelling (Supplemental Table 4).

### Walnut

Twenty-five walnut-induced anaphylaxis cases were reported, 60% (N = 15) in adults and 40% (N = 10) in adolescents (Table 1). Grade 2 reactions were observed in 40% of adolescents and 53.3% of adults, grade 3 in 60% and 40%, respectively, and 1 grade 4 reaction (6.7%) occurred in adults, which was fatal. Cofactors were common, reported in 73.3% of adult and 50% of adolescents (Supplemental Table 8). PE was the predominant cofactor in adolescents (40%), whereas adults exhibited a broader range of cofactors (Supplemental Fig. 4B). Previous walnut-allergy diagnosis was more frequent in adolescents (62.5%) compared to adults (15.4%; *P* = 0.056) (Supplemental Table 8). Most reactions (92.3%) followed ingestion of small amounts of typically native walnuts (<1 teaspoon–1 tablespoon; Supplemental Table 3). In all cases except 1 involving unrecognized exposure in a salad, walnut was easily identifiable (Supplemental Table 4).

### Almond

Twenty-four almond-induced anaphylaxis cases were reported, mainly in adults (N = 20; 83.3%), with few cases in adolescents (N = 4; 16.7%; Table 1). Grade 2 reactions were observed in 25% of adolescents vs 55% of adults, and grade 3 in 75% vs 45%, respectively. No grade 4 reactions or fatalities were reported. Cofactors were frequent, reported in 2 adolescent cases (66.7%, both PE-associated) and in 60% of adult cases, mostly multiple cofactors (33.3%) and alcohol (25%) (Supplemental Table 8; Supplemental Fig. 4D). BPA and OAS were each seen in 2 adolescents (66.7%). In adults, BPA was reported in 7 (38.9%) and OAS in 4 (20%) individuals (Supplemental Table 8). Reactions occurred across a wide range of exposure amounts (<1 teaspoon to 1/2 cup; Supplemental Table 3). Almond was identifiable in all but 1 case in which the patient was unaware that the consumed macarons contained almond (Supplemental Table 4).

## Discussion

To our knowledge, this is the first study to investigate FIA attributed to plant-proteins recommended for individuals seeking to adopt PBDs (plant allergens; PAs) using data from a large cohort of FIA collected by the AVN over a 23-year period (2002–2024). Although prior publications have utilized the same dataset, this work focuses exclusively on PAs and provides a novel characterization of clinical phenotypes in adolescents and adults.^21^^,^^32^^,^^33^

Interestingly, our results showed that the total PA-related FIA cases remained stable in adults, whereas in adolescents they increased significantly after approximately 2017. The reasons for this increase are unclear. It may reflect changing dietary patterns in this age group driven by ethical and environmental concerns.^34^ Policy measures such as the French legislation mandating a weekly vegetarian meal in school canteens could be a contributing factor by increasing awareness of PBDs among adolescents.^35^ However a direct link between such policies and FA has not been established. Alternatively, the increase might partly reflect case-recording biases inherent to registry-based data, including changes in reporting practices over time.

In our study, eleven allergens accounted for 85% of all PA-related FIA cases. Wheat-induced FIA in this cohort followed a recognizable, well-described clinical phenotype. Wheat was the leading elicitor, particularly in early and middle-aged adults, and reactions were modulated by cofactors, consistent with wheat dependent exercise-induced anaphylaxis.^15^^,^^36^^,^^37^ While PE remained the predominant cofactor in adolescents, combination of cofactors, including alcohol and medication use were common in adults. Atopic comorbidities were uncommon but more frequent in adolescents, than in adults, aligning with prior age-related observations.^36^ Recurrent reactions, including urticaria or anaphylaxis, were common in both age groups and appears to be an important diagnostic consideration. Sensitization to ω-5 gliadin predominated across both age groups (CRD available for 60% of cases in both age groups), reinforcing its role as a key molecular marker of wheat-induced anaphylaxis. Additionally, isolated sensitization to lipid transfer protein (Tri a 14) was also observed in a few cases, particularly among adolescents consistent with previous reports describing Tri a 14 sensitization in younger patients.^38^

Within the legume family, peanut-induced anaphylaxis in our cohort followed a well-established age-dependent phenotype, occurring predominantly in adolescents and young adults with a pre-existing peanut-allergy.^15^^,^^39^ Soy is a major ingredient in plant-based foods due to its high protein content.^40^ Consistent with previous reports, soy-induced anaphylaxis in adults was associated with BPA, PR-10 sensitization (Gly m 4, Bet v 1), and ingestion of large quantities, most commonly soy beverages.^15^^,^^41^ Our data extends these findings by identifying a possible adolescent phenotype. This phenotype was characterized by reactions in the context of pre-existing legume-allergy and a relative predominance of sensitization to seed storage proteins (Gly m 5 and Gly m 6) which may suggest cross-reactivity. However, given the small adolescent sample size and the incomplete CRD data, this age-specific phenotype would need confirmation in future AVN data. Lupin also emerged as a frequent elicitor of FIA, particularly in adults.^42^, ^43^, ^44^, ^45^, ^46^ In our cohort, lupin-induced FIA showed a female predominance in adults. In adolescents it occurred predominantly in those with pre-exisitng legume allergy. This female predominance warrants confirmation in larger studies. Possible contributing factors include sex-hormone influences, greater PBD adoption among women leading to differential dietary exposure, and cutaneous sensitization via lupin-derived ingredients in dermocosmetic products, though these remain unproven.^47^, ^48^, ^49^ Other legumes used in PBDs, such as pea and lentil, were less frequently implicated in anaphylaxis among both adolescents and adults. This is consistent with the currently available evidence on pea and lentil allergy, which is mostly reported in pediatric age groups.^50^, ^51^, ^52^ Their lower frequency as triggers of FIA in older age groups suggests that peas and lentils may represent relatively lower-risk plant protein options from adolescence onwards. 50, 51, 52

Buckwheat, a gluten-free pseudocereal increasingly used in Europe as a sustainable alternative to traditional cereals, is an emerging allergen.^32^^,^^53^, ^54^, ^55^ Buckwheat-induced anaphylaxis was more frequently reported in middle-aged adults than in adolescents. Aside from a higher proportion of adolescents with pre-existing buckwheat-allergy, no other major differences were observed between the 2 age groups. Overall, no clear association with cofactors or BPA was identified, and reactions can occur at both small-large quantities, with no distinct clinical phenotype emerging.

Among tree nuts relevant to PBDs, hazelnut was a common FIA elicitor in both age groups with no clear age-specific clinical differences. Reactions were often cofactor-associated, and BPA was common, consistent with birch pollen-driven sensitization via PR-10.^56^ Whereas earlier studies reported cashew allergy predominantly in children,^15^^,^^57^ our data extend these findings by showing that in adolescents cashew-induced anaphylaxis mainly occurred in individuals with pre-existing cashew allergy, while in adults reactions were more often associated with cofactors. Walnut-induced anaphylaxis showed no distinct age-specific patterns apart from higher rates of pre-existing allergy in adolescents. Across both age groups, cofactors were frequently implicated. Consistent with previous reports, almond-induced anaphylaxis occurred predominantly in adults, typically following ingestion of larger amounts.^57^

Seeds have gained prominence due to their high nutritional value.^58^ Among them, sesame is the most frequent elicitor of FIA across both adolescents and middle-aged adults, with no clear clinical phenotype. Cofactors were involved in approximately one-third of cases, while pre-existing sesame-allergy was more common in adolescents. Pine nut is the second most frequent seed-related elicitor of FIA, affecting adolescents and young adults at similar rates. Cofactor involvement, mainly alcohol, was more often reported in adults.

Most reactions followed exposure to small amounts (<1 teaspoon–1 tablespoon), primarily involving peanut, tree nuts, sesame, pine nut. Reactions to unidentified allergen were reported in up to 22.3% of peanut, lupin (19%), pine nut (19.2%), buckwheat (16.7%) and sesame (14.6%) cases. Particular attention is warranted for pine nut and buckwheat in absence of mandatory allergen labeling in the European Union.^59^

Adoption of PBDs in food-allergic or atopic individuals may benefit from consideration of age-specific clinical patterns. In adults, BPA was common among FIA involving soy typically following larger exposures. Hazelnut and almond-related FIA in adults were also frequently associated with BPA and could be triggered by small to large exposures. Wheat was the most frequent elicitors in adults. Across these PA-related FIA, reactions commonly occurred in the presence of diverse cofactors, often combined, including alcohol, medications and PE. In adolescents, PE was typically the main cofactor in most of the PA-related FIA cases. Peanut, cashew, walnut, sesame and buckwheat anaphylaxis more often occurred in the context of pre-existing allergy to the culprit allergen. Soy and lupin anaphylaxis occurred commonly in individuals with a known legume-allergy. Similar to adults, hazelnut reactions were common in individuals with BPA. Clinical phenotype of wheat-related reactions were similar to adults.

For sesame, pine nut, and buckwheat heterogeneous clinical presentations limited the ability to draw age-specific phenotypes.

Thus, distinct clinical patterns emerged across certain PA triggers of FIA, allowing differentiation between allergens with age-specific clinical phenotypes and those with heterogeneous presentations. This distinction is clinically relevant, as it provides a framework for dietary counseling in food-allergic or atopic adolescents and adults considering adoption of PBDs (Fig. 5).

## Limitations

This study has limitations. The AVN relies on voluntary reporting of FIA, which does not allow prevalence estimation. Dietary habits were not collected; findings are not specific to individuals following a PBD. In addition, CRD were not systematically available for all cases, partly due to the long study period beginning in 2002, prior to the widespread use of CRD in routine clinical practice in France. Analyses were bivariate; as cofactors, comorbidities, prior diagnosis, and age are likely interrelated, thus findings represent associations rather than independent effects. Finally, some variables contained missing data, and analyses were therefore based on available observations.

## Conclusion

FIA due to certain PAs exhibit distinct age-specific clinical phenotypes. Improved awareness of these patterns may help guide dietary counseling for food-allergic or atopic individuals considering adopting more PBDs. Future prospective, population-based studies with diet-adherence data are warranted to evaluate the relationship between PBD adoption and FA risk. Anaphylaxis registries have a key role in this perspective and should now capture dietary information to support such analyses.

## Disclosure of the use of generative AI and AI-assisted technologies

The authors used ChatGPT (OpenAI) solely to improve the readability and language of the manuscript. All scientific content, interpretations, conclusions, and figures were developed and verified independently by the authors without the use of AI.

## Funding information

Supported by the Luxembourg National Research Fund on PRIDE program grant PRIDE i2TRON PRIDE19/14254520 and the Marie Sklodowska-Curie Grant Agreement No. 101072377 by the European Union's 10.13039/100018693Horizon Europe Research and Innovation Program. Supported additionally by the 10.13039/100009647Ministry of Health and the 10.13039/501100004562Ministry of Higher Education and Research.

## Conflict of interest

GP declares that he has received fees for scientific work or consulting requested by Viatris, Novartis, DVB Technology, Bioprojet, Stallergenes.

ADC, outside of the submitted work, reports grants from Don du Souffle, Novartis, ARAIRLOR, French Academy of Medicine, French Society of Paediatric Pulmonology and Allergology, consulting fees from Sanofi, Stallergens, ALK, Aimmune Therapeutics, payment for presentations for Aimmune Therapeutics, Novartis, ALK, DBV, support for attending meetings from Mead Johnson, Nutricia, Aimmune Therapeutics, Novartis, ALK, DBV, stocks from Essilor Luxottica.

The other authors declare that they have no conflicts of interest relevant to this work.

## Figures and Tables

**figs1:**
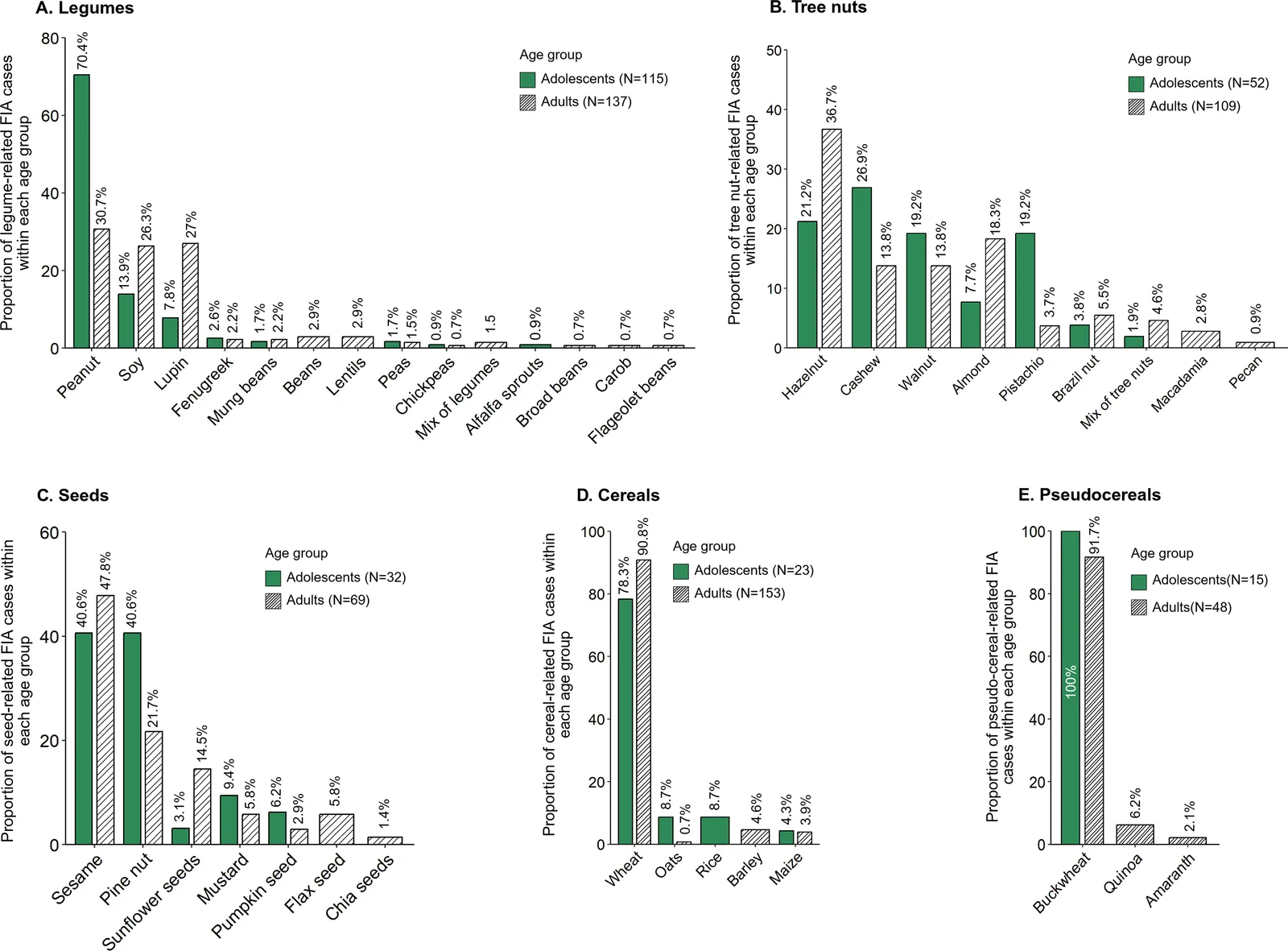
Proportion of food-induced anaphylaxis (FIA) attributable to individual allergens within plant-based diet allergen (PBD) families in adolescents and adults. Panels shows causative allergens within (A) legumes (B) tree nuts (C) seeds (D) cereals and (E) pseudocereals family. N indicates the total number of FIA cases per PBD allergen family for adolescents and adults. (Percentages are calculated using the total number of FIA cases in each PBD allergens family for adolescents and adults respectively).

**figs2:**
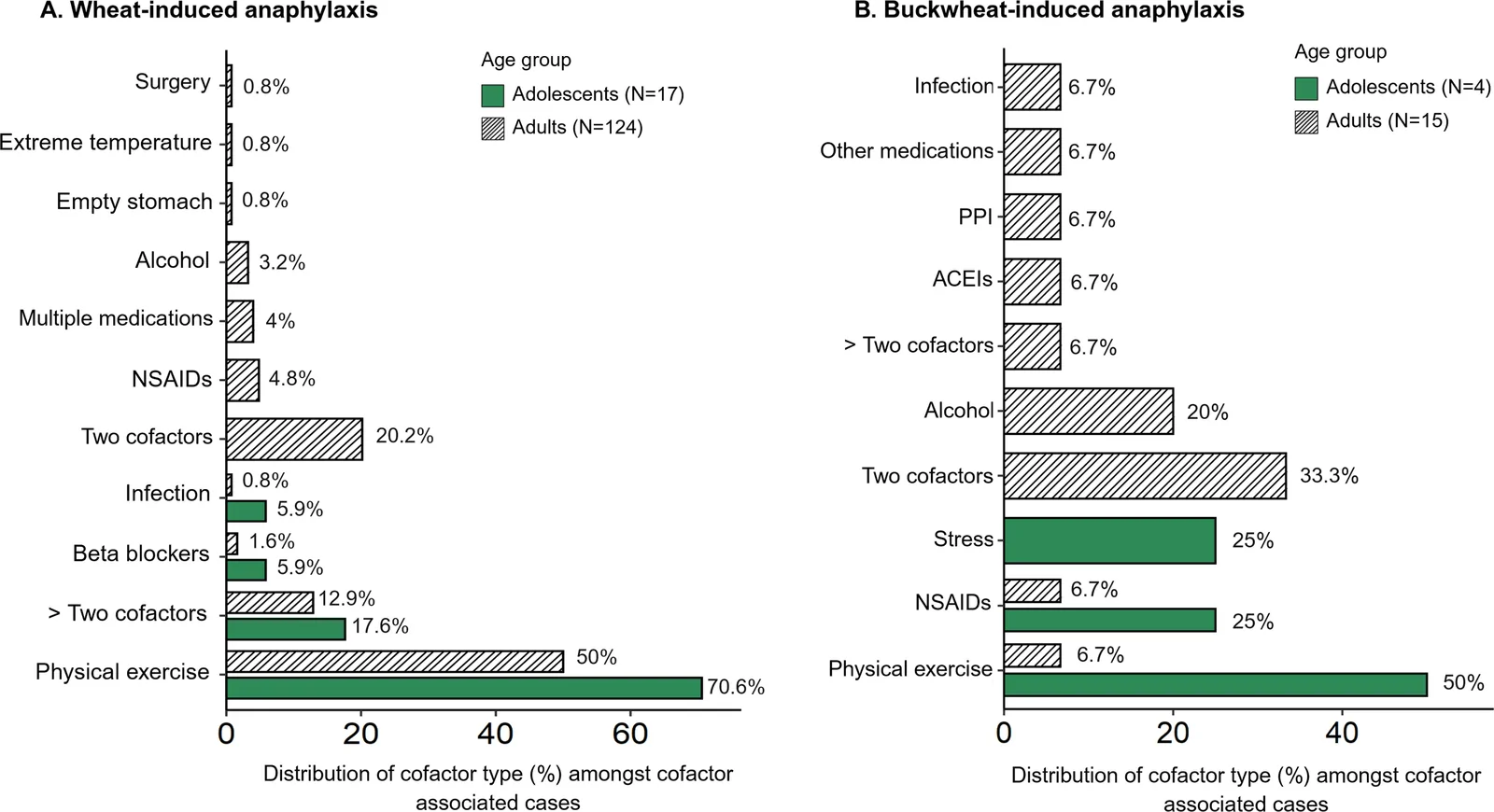
Types of cofactors associated with food-induced anaphylaxis (FIA) in adolescents and adults, attributed to (A) wheat-induced anaphylaxis and (B) buckwheat-induced anaphylaxis. N indicates the total number of FIA cases associated with a cofactor within each age group. “Other medications” refers to drugs other than non-steroidal anti-inflammatory drugs (NSAIDs), angiotensin-converting enzyme inhibitors (ACEIs), proton pump inhibitors (PPI), and beta-blockers.

**figs3:**
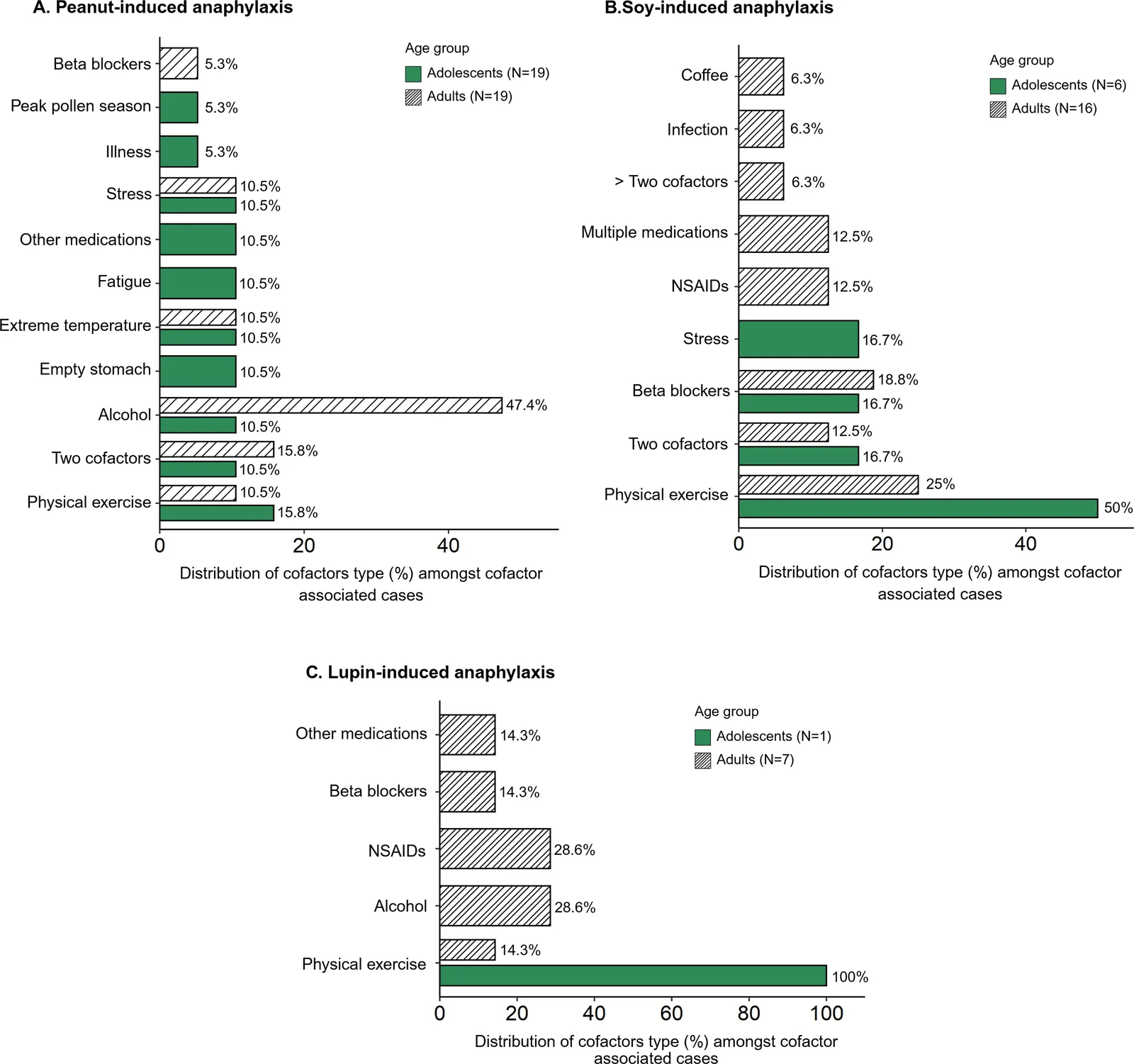
Types of cofactors associated with food-induced anaphylaxis (FIA) in adolescents and adults, attributed to (A) peanut-induced anaphylaxis, (B) soy-induced anaphylaxis and (C) lupin-induced anaphylaxis. N indicates the total number of FIA cases associated with a cofactor within each age group. “Other medications” refers to drugs other than non-steroidal anti-inflammatory drugs (NSAIDs), angiotensin-converting enzyme inhibitors (ACEIs), proton pump inhibitors (PPI), and beta-blockers.

**figs4:**
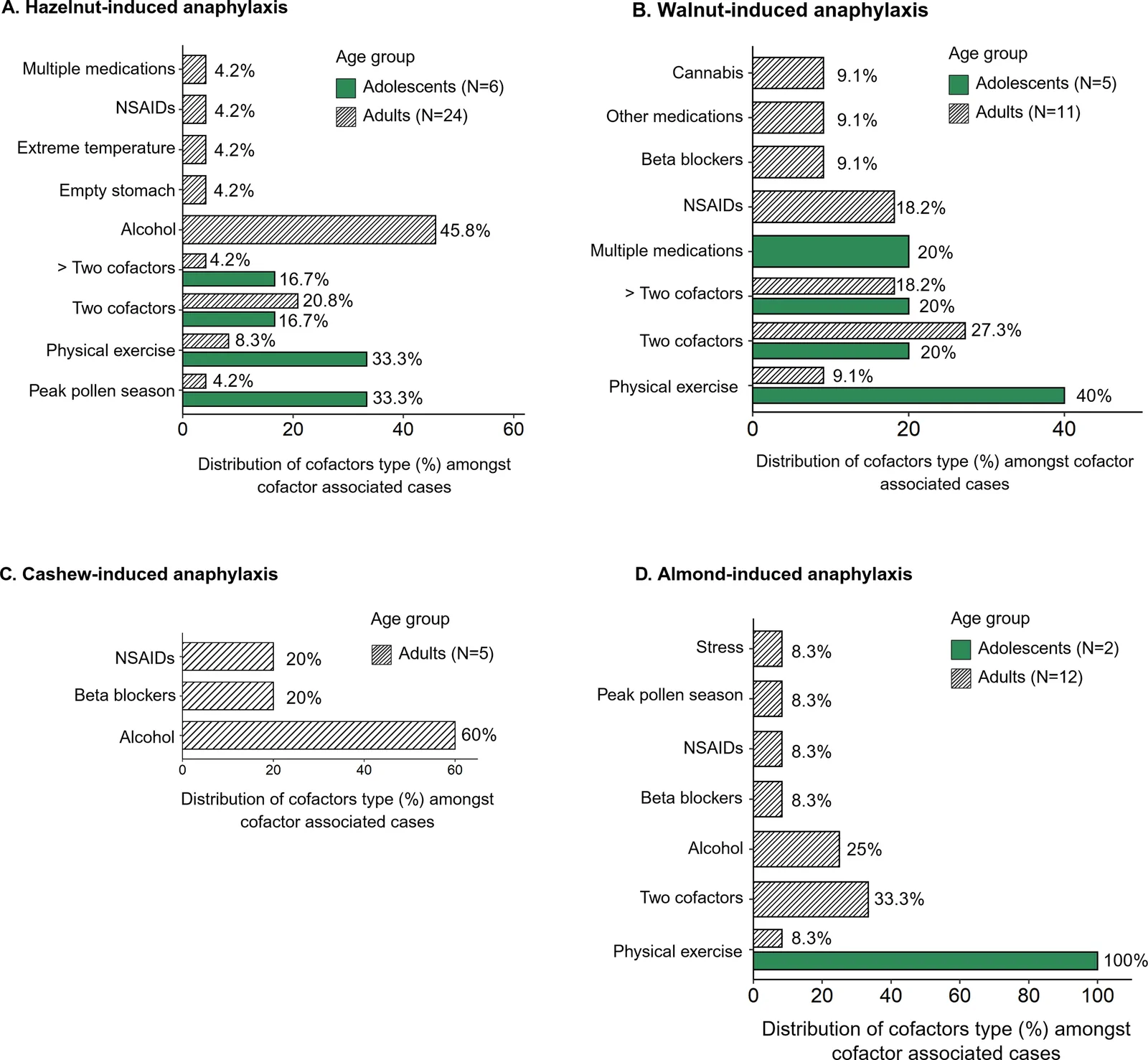
Types of cofactors associated with food-induced anaphylaxis (FIA) in adolescents and adults, attributed to (A) hazelnut-induced anaphylaxis, (B) walnut-induced anaphylaxis (C) cashew-induced anaphylaxis, and (D) almond-induced anaphylaxis. N indicates the total number of FIA cases associated with a cofactor within each age group. “Other medications” refers to drugs other than non-steroidal anti-inflammatory drugs (NSAIDs), angiotensin-converting enzyme inhibitors (ACEIs), proton pump inhibitors (PPI), and beta-blockers.

**figs5:**
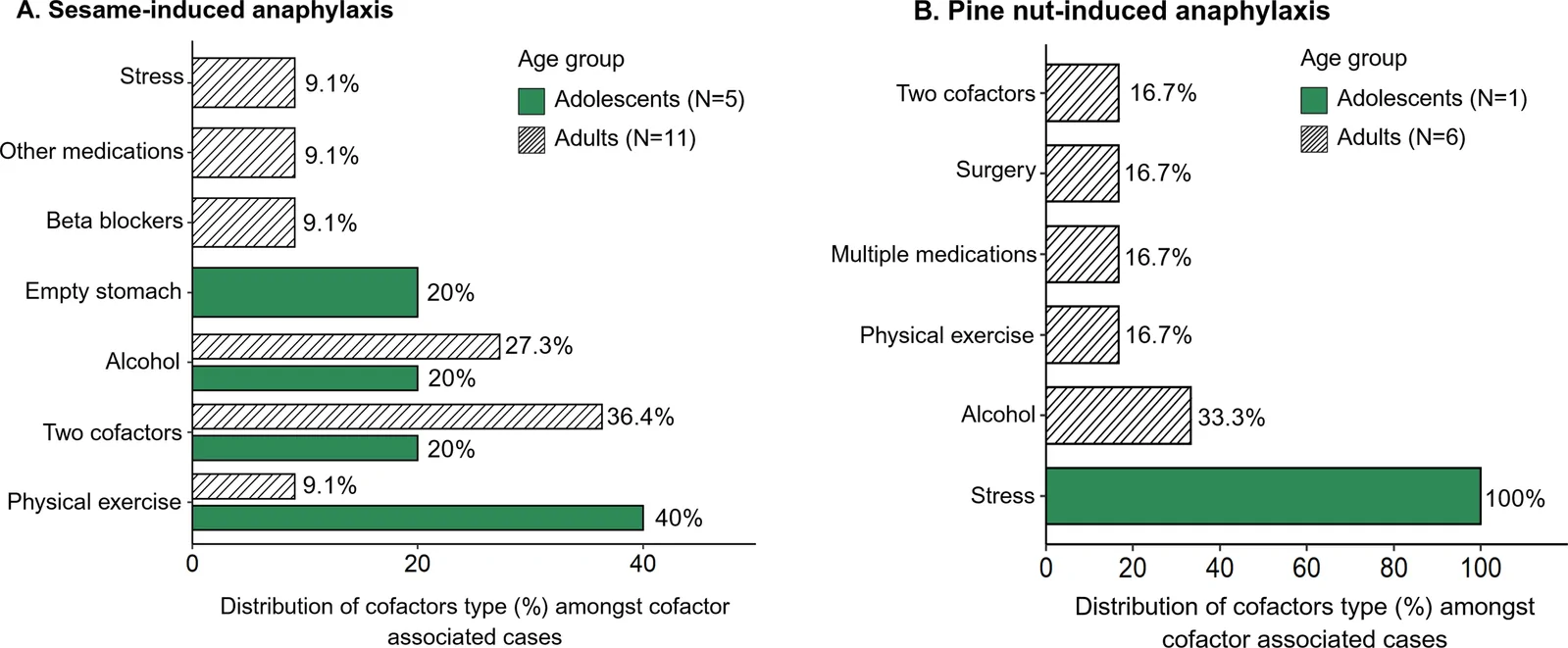
Types of cofactors associated with food-induced anaphylaxis (FIA) in adolescents and adults, attributed to (A) sesame-induced anaphylaxis, (B) pine nut-induced anaphylaxis. N indicates the total number of FIA cases associated with a cofactor within each age group. “Other medications” refers to drugs other than non-steroidal anti-inflammatory drugs (NSAIDs), angiotensin-converting enzyme inhibitors (ACEIs), proton pump inhibitors (PPI), and beta-blockers.
