# Supplementary material for: Food-induced anaphylaxis to plant allergens in adolescents and adults: Data from the allergy Vigilance Network (2002–2024)
Source: World Allergy Organ J. 2026 Aug 13;19(8):101448. doi: 10.1016/j.waojou.2026.101448 (PMC13499346; doi:10.1016/j.waojou.2026.101448)
Supplement: Supplementary file 1 [file mmc1.docx]

**Supplemental Table 1 Definitions of the various plant-based diets**

| ***Main category*** | |
| --- | --- |
| **Plant-based diet** | Prioritize foods derived primarily from plants while allowing for the optional inclusion of small amounts of animal products |
| ***Subcategories*** | |
| **Flexitarian** | Primarily vegetarian but include meat, dairy, eggs, poultry and fish on occasion, or in small quantities. |
| **Pescatarian** | Includes plant derived food + seafood + dairy + egg but excludes red meat and poultry |
| **Lacto-ovo-vegetarian** | Includes plant derived food + dairy + eggs but excludes red meat, poultry and seafood |
| **Lacto-vegetarian** | Includes plant derived food + dairy products such as milk, cheese, butter, yoghurt, cream etc. but excludes red meat, poultry, eggs and seafood |
| **Ovo-vegetarian** | Includes plant derived food + eggs but excludes dairy, red meat, poultry, and seafood |
| **Vegan** | Includes only plant derived food and excludes as animal products |

Definitions adapted from the World Health Organization. (1)

**Supplemental Table 2 Clinical phenotypes of food-induced anaphylaxis attributed to main cereal allergen in adolescents vs. adults**

| **Wheat**  **Total number of cases reported to AVN =157** | | | |
| --- | --- | --- | --- |
| **Clinical characteristics** | **Adolescents** | **Adults** | **P value** |
| Number of cases; N (%) | 18 (11.5) | 139 (88.5) | - |
| Median age in years; IQR | 17; 15.2-17.8 | 41; 33-54 | **<0.001** |
| Male; N (%) | 8 (44) | 79 (56.8) | 0.457 |
| Severity of reactions; N (%)  Grade 2  Grade 3  Grade 4 | 12 (66.7)  6 (33.3)  0 | 69 (49.6)  67 (48.2)  3 (2.2) | 0.461 |
| Cofactors; N (%)  *Data available (N)* | 17 (94.4)  *(N=18)* | 124 (90.5)  *(N=137)* | 1.000 |
| **Atopic comorbidities; N (%)** | | | |
| Asthma  *Data available (N)* | 5 (29.4)  *(N=17)* | 12 (9)  *(N=133)* | **0.027** |
| Birch pollen allergy  *Data available (N)* | 2 (11.8)  *(N=17)* | 7 (5.4)  *(N=129)* | 0.282 |
| Prior allergy diagnosis to culprit allergen  *Data available (N)* | 1 (5.9)  *(N=17)* | 14 (10.9)  *(N=129)* | 1.000 |
| Previous allergic symptoms with the same allergen;  *Data available (N)*  -Recurrent urticaria  -Recurrent angioedema  - Anaphylaxis  -Local symptoms (pruritus or OAS) | 11 (64.7)  *(N=17)*  2 (11.8)  -  9 (52.9)  - | 79 (60.3)  *(N=131)*  20 (15.3)  2 (1.5)  55 (42)  2 (2.5) | 0.932 |
| **Wheat component-resolved diagnostics (CRD)^a^; N (%)** | | | |
| Tri a 19 (Omega-5-Gliadin)  Tri a 14 (nsLTP)  *Data available (N)* | 8 (72.7)  3 (27.3)  *(N=11)* | 85 (97.7)  2 (2.3)  *(N=87)* | **0.009** |

Data were complete for all cases for age, sex, and severity of reactions. CRD were available for a subset of cases (adolescents N=11, 61.1%; adults N=87, 62.6%). "Data available (N)" denotes the number of cases with non-missing data for that variable. Percentages are calculated using available data as the denominator (complete-case analysis, no imputation). AVN, Allergy-Vigilance Network; IQR, Interquartile range (25^th^ and 75^th^ percentile); nsLTP, Non-specific lipid transfer protein; OAS, Oral allergy syndrome; ^a^Values as measured on ImmunoCAP, Thermo Fisher Scientific.

**Supplemental Table 3 Eliciting doses of the main plant-based diet allergens in adolescents and adults**

| **Allergens** | **Total cases reported to AVN** | **Data available** | **Contact or Inhalation** | **<1 teaspoon** | **1 teaspoon** | **1 tablespoon** | **½ cup** | **1 cup** | **1 plate or full serving** |
| --- | --- | --- | --- | --- | --- | --- | --- | --- | --- |
| **Wheat** | 157 | 38 | - | 2 (5.3%) | - | 3 (7.9%) | 3 (7.9%) | - | 30 (78.9%) |
| **Peanut** | 123 | 51 | 2 (3.9%) | 28 (54.9%) | 4 (7.8%) | 4 (7.8%) | 5 (9.8%) | - | 8 (15.7%) |
| **Buckwheat** | 59 | 39 | - | 9 (23.1%) | 1 (2.6%) | 2 (5.1%) | 3 (7.7%) | 1 (2.6%) | 23 (59%) |
| **Soy** | 52 | 28 | - | 5 (17.9%) | 2 (7.1%) | 4 (14.3%) | 3 (10.7%) | 11 (39.3%) | 3 (10.7%) |
| **Hazelnut** | 51 | 34 | - | 14 (41.2%) | 4 (11.8%) | 6 (17.6%) | 7 (20.6%) | 1 (2.9%) | 2 (5.9%) |
| **Lupin** | 46 | 12 | - | 1 (8.3%) | 1 (8.3%) | 1 (8.3%) | 1 (8.3%) | - | 8 (66.7%) |
| **Sesame** | 46 | 10 | - | 2 (20%) | 1 (10%) | 3 (30%) | - | - | 4 (40%) |
| **Cashew** | 29 | 16 | - | 8 (50%) | 2 (12.5%) | 3 (18.8%) | 1 (6.2%) | - | 2 (12.5%) |
| **Pine nut** | 28 | 14 | - | 10 (71.4%) | - | 1 (7.1%) | - | - | 3 (21.4%) |
| **Walnut** | 25 | 13 | - | 5 (38.5%) | 2 (15.4%) | 5 (38.5%) | - | - | 1 (7.7%) |
| **Almond** | 24 | 12 | - | 4 (33.3%) | - | 4 (33.3%) | 4 (33.3%) | - | - |

Percentages are calculated using available data as the denominator (complete-case analysis, no imputation). AVN; Allergy-Vigilance Network

**Supplemental Table 4 Hidden vs. identifiable allergens in the food products consumed prior to reaction (adolescents and adults)**

| **Allergens** | **Total number of cases reported** | **Number of cases with available data**  **(N)** | **Allergen identifiable**  **N (%)^a^** | **Allergen non-identifiable**  **N (%)^a^** |
| --- | --- | --- | --- | --- |
| **Wheat** | 157 | 146 | 141 (96.6) | 5 (3.4) |
| **Peanut** | 123 | 112 | 87 (77.7) | 25 (22.3) |
| **Buckwheat** | 59 | 54 | 45 (83.3) | 9 (16.7) |
| **Soy** | 52 | 47 | 45 (95.7) | 2 (4.3) |
| **Hazelnut** | 51 | 48 | 45 (93.8) | 3 (6.2) |
| **Lupin** | 46 | 42 | 34 (81) | 8 (19) |
| **Sesame** | 46 | 41 | 35 (85.4) | 6 (14.6) |
| **Cashew** | 29 | 28 | 27 (96.4) | 1 (3.6) |
| **Pine nut** | 28 | 26 | 21 (80.8) | 5 (19.2) |
| **Walnut** | 25 | 23 | 22 (95.7) | 1 (4.3) |
| **Almond** | 24 | 23 | 22 (95.7) | 1 (4.3) |

^a^Percentages are calculated using available data as the denominator (complete-case analysis, no imputation).

**Supplemental Table 5 Clinical phenotypes of food-induced anaphylaxis attributed to main legume allergens in adolescents vs. adults**

| **Peanut**  **Total number of cases reported to AVN = 123** | | | |
| --- | --- | --- | --- |
| **Clinical characteristics** | **Adolescents** | **Adults** | **P value** |
| Number of cases; N (%) | 81 (65.9) | 42 (34.1) |  |
| Median age in years; IQR | 16; 14-17 | 25.5; 22.2-34.8 | **<0.001** |
| Male; N (%) | 38 (46.9) | 24 (57.1) | 0.343 |
| Severity of reactions; N (%)  Grade 2  Grade 3  Grade 4 | 48 (59.3)  25 (30.9)  8 (9.9) | 25 (59.5)  15 (35.7)  2 (4.8) | 0.641 |
| Cofactors; N (%)  *Data available (N)* | 19 (25.3)  *(N=75)* | 19 (50)  *(N=38)* | **0.016** |
| **Atopic comorbidities; N (%)** | | | |
| Asthma  *Data available (N)* | 39 (52)  *(N=75)* | 14 (35.9)  *(N=39)* | 0.151 |
| Birch pollen allergy  *Data available (N)* | 10 (14.5)  *(N=69)* | 9 (25)  *(N=36)* | 0.289 |
| Prior allergy diagnosis to culprit allergen  *Data available (N)* | 56 (71.8)  *(N=78)* | 18 (46.2)  *(N=39)* | **0.012** |
| Prior other legume-allergy diagnosis  *Data available (N)* | 12 (16)  *(N=75)* | 5 (12.8)  *(N=39)* | 0.785 |
| History of OAS  *Data available (N)* | 11 (14.7)  (N=75) | 6 (15.4)  *(N=39)* | 1.000 |
| **Soy**  **Total number of cases reported to AVN = 52** | | | |
| **Clinical characteristics** | **Adolescents** | **Adults** | **P value** |
| Number of cases; N (%) | 16 (30.8) | 36 (69.2) | - |
| Median age in years; IQR | 15; 14.8-16.2 | 46; 34.8-60.2 | **<0.001** |
| Male; N (%) | 7 (43.8) | 12 (33.3) | 0.683 |
| Severity of reactions; N (%)  Grade 2  Grade 3  Grade 4 | 13 (81.2)  3 (18.8)  0 | 29 (80.6)  7 (19.4)  0 | 1.000 |
| Cofactors; N (%)  *Data available (N)* | 6 (37.5)  *(N=16)* | 16 (44.4)  *(N=36)* | 0.765 |
| **Atopic comorbidities; N (%)** | | | |
| Asthma  *Data available (N)* | 6 (37.5)  *(N=16)* | 8 (23.2)  *(N=34)* | 0.330 |
| Birch pollen allergy  *Data available (N)* | 3 (18.8)  *(N=16)* | 20 (64.5)  *(N=31)* | **0.005** |
| Bet v 1 sensitization^‡^  *Data available (N)* | 4 (25)  *(N=16)* | 17 (54.8)  *(N=31)* | 0.068 |
| Prior allergy diagnosis to culprit allergen  *Data available (N)* | 1 (7.7)  *(N=13)* | 4 (13.8)  *(N=29)* | 1.000 |
| Prior peanut or other legume allergy diagnosis  *Data available (N)* | 8 (50)  *(N=16)* | 2 (5.6)  *(N=36)* | **<0.001** |
| History of OAS  *Data available (N)* | 5 (31.2)  *(N=16)* | 17 (53.1)  *(N=32)* | 0.221 |
| **Soy component-resolved diagnostics (CRD)^a^; N (%)** | | | |
| Gly m 4 (PR-10 related)  Gly m 5 or Gly m 6 or both (Both globulin)  Gly m 4, 5, 6  *Data available (N)* | 5 (45.5)  5 (45.5)  1 (9.1)  *(N=11)* | 21 (84)  1 (4)  3 (12)  *(N=25)* | 0.059^b^  **0.018^b^**  1.000^b^ |
| **Lupin**  **Total number of cases reported to AVN = 46** | | | |
| **Clinical characteristics** | **Adolescents** | **Adults** | **P value** |
| Number of cases; N (%) | 9 (19.6) | 37 (80.4) | - |
| Median age in years; IQR | 15; 13-18 | 49; 31-60 | **<0.001** |
| Male; N (%) | 4 (44.4) | 7 (18.9) | 0.186 |
| Severity of reactions; N (%)  Grade 2  Grade 3  Grade 4 | 9 (100)  0  0 | 30 (81.1)  7 (18.9)  0 | 0.316 |
| Cofactors; N (%)  *Data available (N)* | 1 (12.5)  *(N=8)* | 7 (20.6)  *(N=34)* | 1.000 |
| **Atopic comorbidities; N (%)** |  |  |  |
| Asthma  *Data available (N)* | 1 (12.5)  *(N=8)* | 9 (29)  *(N=31)* | 0.653 |
| Birch pollen allergy  *Data available (N)* | 0  *(N=8)* | 0  *(N=30)* | **-** |
| Prior allergy diagnosis to culprit allergen  *Data available (N)* | 3 (28.6)  *(N= 7)* | 20 (33.3)  *(N=15)* | 0.639 |
| Prior peanut or other legume allergy diagnosis  *Data available (N)* | 3 (37.5)  *(N=8)* | 3 (9.4)  *(N=32)* | 0.082 |
| History of OAS  *Data available (N)* | 0  *(N=8)* | 2 (6.2)  *(N=32)* | 1.000 |

Data were complete for all cases for age, sex, and severity of reactions. Soy CRD were available for a subset of cases (adolescents N=11, 68.8%; adults N=87, 69.4%). "Data available (N)" denotes the number of cases with non-missing data for that variable. Percentages are calculated using available data as the denominator (complete-case analysis, no imputation. AVN, Allergy-Vigilance Network; CRD, Component-resolved diagnostics; IQR, Interquartile range (25^th^ and 75^th^ percentile); OAS, Oral allergy syndrome; ^a^Values as measured on ImmunoCAP, Thermo Fisher Scientific; ^b^Adjusted p value using Benjamini–Hochberg correction.

**Supplemental Table 6 Clinical phenotypes of food-induced anaphylaxis attributed to main pseudocereal allergen in adolescents vs. adults**

| **Buckwheat**  **Total number of cases reported to AVN =59** | | | |
| --- | --- | --- | --- |
| **Clinical characteristics** | **Adolescents** | **Adults** | **P value** |
| Number of cases; N (%) | 15 (25.4) | 44 (74.6) | - |
| Median age in years; IQR | 15; 13-16.5 | 37.5; 30-49 | **<0.001** |
| Male; N (%) | 6 (40) | 21 (47.7) | 0.827 |
| Severity of reactions; N (%)  Grade 2  Grade 3  Grade 4 | 11 (73.3)  4 (26.7)  0 | 27 (61.4)  17 (38.6)  0 | 0.537 |
| Cofactors; N (%)  *Data available (N)* | 4 (26.7)  *(N=15)* | 15 (35.7)  *(N=42)* | 0.751 |
| **Atopic comorbidities; N (%)** |  |  |  |
| Asthma  *Data available (N)* | 8 (53.3)  *(N=15)* | 16 (42.1)  *(N=38)* | 0.665 |
| Birch pollen allergy  *Data available (N)* | 1 (7.1)  *(N=14)* | 1 (3)  *(N=33)* | 0.512 |
| Prior allergy diagnosis to culprit allergen  *Data available (N)* | 7 (46.7)  *(N=15)* | 5 (13.9)  *(N=36)* | **0.026** |

Data were complete for all cases for age, sex, and severity of reactions. "Data available (N)" denotes the number of cases with non-missing data for that variable. Percentages are calculated using available data as the denominator (complete-case analysis, no imputation. AVN, Allergy-Vigilance Network; IQR, Interquartile range (25^th^ and 75^th^ percentile); OAS, Oral allergy syndrome

**Supplemental Table 7 Type of food item consumed during soy-induced anaphylaxis**

| **Stratified by age group** | | |
| --- | --- | --- |
|  | **Adolescents**  **N (%)** | **Adults**  **N (%)** |
| *Data available on food product consumed* | 16 | 34 |
| Type of food product consumed prior to reaction | | |
| **Soy beverages** | 6 (37.5) | 24 (70.6) |
| **Soy based dessert** | **-** | 5 (14.7) |
| **Meat preparation** | 3 (18.8) | - |
| **Meat alternative** | 2 (12.5) | - |
| **Processed soy products**  **(soy sauce, miso, tofu)** | 2 (12.5) | 3 (8.8) |
| **Soy oil** | 1 (6.2) | - |
| **Soybeans** | 1 (6.2) | 2 (5.9) |
| **Contamination** | 1 (6.2) | - |
| **Stratified by component allergen sensitization** | | |
|  | **Gly m 5 and/or Gly m 6 (Globulins)** | **Gly m 4 (PR-10 related protein)** |
| *Data available on food product consumed* | 6 | 24 |
| Type of food product consumed prior to reaction | | |
| **Soy beverages** | 1 (16.7) | 12 (50) |
| **Soy based dessert** | - | 5 (20.8) |
| **Meat preparation** | 1 (16.7) | 1 (4.2) |
| **Meat alternative** | 1 (16.7) | 1 (4.2) |
| **Processed soy products**  **(soy sauce, miso, tofu)** | - | 4 (16.7) |
| **Soy oil** | 1 (16.7) | - |
| **Soybeans** | 2 (33.3) | 1 (4.2) |

Percentages are calculated using available data as the denominator (complete-case analysis, no imputation).

**Supplemental Table 8 Clinical phenotypes of food-induced anaphylaxis attributed to main tree nut allergen in adolescents vs. adults**

| **Hazelnut**  **Total number of cases reported to AVN = 51** | | | |
| --- | --- | --- | --- |
| **Clinical characteristics** | **Adolescents** | **Adults** | **P value** |
| Number of cases; N (%) | 11 (21.6) | 40 (78.4) |  |
| Median age in years; IQR | 16; 13.8-16.5 | 44.5; 30.8-54.2 | **<0.001** |
| Male; N (%) | 6 (54.5) | 20 (50) | 1.000 |
| Severity of reactions; N (%)  Grade 2  Grade 3  Grade 4 | 7 (63.6)  4 (36.4)  0 (0) | 26 (65)  12 (30)  2 (5) | 1.000 |
| Cofactors; N (%)  *Data available (N)* | 6 (54.5)  *(N=11)* | 24 (63.2)  *(N=38)* | 0.729 |
| **Atopic comorbidities; N (%)** | | | |
| Asthma  *Data available (N)* | 4 (40)  *(N=10)* | 9 (23.7)  *(N=38)* | 0.425 |
| Birch pollen allergy  *Data available (N)* | 6 (54.5)  *(N=11)* | 12 (34.3)  *(N=35)* | 0.296 |
| Bet v 1 sensitization^a^  *Data available (N)* | 5 (100)  *(N=5)* | 9 (81.8)  *(N=11)* | 0.278 |
| Prior allergy diagnosis to culprit allergen  *Data available (N)* | 4 (40)  *(N=10)* | 5 (14.7)  *(N=34)* | 0.175 |
| History of OAS  *Data available (N)* | 5 (50)  (N=10) | 8 (20.5)  *(N=39)* | 0.104 |
| **Hazelnut component-resolved diagnostics (CRD)^a^; N (%)** | | | |
| Cor a 1 (PR-10 protein)  Cora 1 and Cor a 8 (nsLTP)  Cor a 9 (Globulin) or Cor a 14 (2 S albumin) or both  Cor a 1, 8, 9, 14  *Data available (N)* | 4 (57.1)  1 (14.3)  2 (28.6)  0 (0)  *(N=7)* | 8 (66.7)  3 (25)  0 (0)  1 (8.3)  *(N=12)* | 0.336 |
| **Cashew**  **Total number of cases reported to AVN = 29** | | | |
| **Clinical characteristics** | **Adolescents** | **Adults** | **P value** |
| Number of cases; N (%) | 14 (48.3) | 15 (51.7) | - |
| Median age in years; IQR | 15; 14-16.9 | 35; 35-54 | **<0.001** |
| Male; N (%) | 11 (78.6) | 6 (40) | 0.060 |
| Severity of reactions; N (%)  Grade 2  Grade 3  Grade 4 | 11 (78.6)  3 (21.4)  0 (0) | 8 (53.3)  7 (46.7)  0 (0) | 0.245 |
| Cofactors; N (%)  *Data available (N)* | 0 (0)  *(N=14)* | 5 (38.5)  *(N=13)* | **0.015** |
| **Atopic comorbidities; N (%)** | | | |
| Asthma  *Data available (N)* | 7 (50)  *(N=14)* | 5 (35.7)  *(N=14)* | 0.704 |
| Birch pollen allergy  *Data available (N)* | 2 (15.4)  *(N=13)* | 1 (7.1)  *(N=14)* | 0.596 |
| Prior allergy diagnosis to culprit allergen  *Data available (N)* | 7 (50)  *(N= 14)* | 0 (0)  *(N=13)* | **0.006** |
| History of OAS  *Data available (N)* | 2 (14.3)  *(N=14)* | 1 (7.1)  *(N=14)* | 1.000 |
| **Walnut**  **Total number of cases reported to AVN = 25** | | | |
| **Clinical characteristics** | **Adolescents** | **Adults** | **P value** |
| Number of cases; N (%) | 10 (40) | 15 (60) | - |
| Median age in years; IQR | 15.5; 14.2-16 | 32; 25-43.5 | **<0.001** |
| Male; N (%) | 6 (60) | 6 (40) | 0.428 |
| Severity of reactions; N (%)  Grade 2  Grade 3  Grade 4 | 4 (40)  6 (60)  0 (0) | 8 (53.3)  6 (40)  1 (6.7) | 0.808 |
| Cofactors; N (%)  *Data available (N)* | 5 (50)  *(N=10)* | 11 (73.3)  *(N=15)* | 0.397 |
| **Atopic comorbidities; N (%)** | | | |
| Asthma  *Data available (N)* | 3 (37.5)  *(N=8)* | 3 (21.4)  *(N=14)* | 0.624 |
| Birch pollen allergy  *Data available (N)* | 2 (25)  *(N=8)* | 1 (8.3)  *(N=12)* | 0.537 |
| Prior allergy diagnosis to culprit allergen  *Data available (N)* | 5 (62.5)  *(N=8)* | 2 (15.4)  *(N=13)* | 0.056 |
| History of OAS  *Data available (N)* | 2 (22.2)  *(N=9)* | 1 (7.1)  *(N=14)* | 0.538 |
| **Almond^b^**  **Total number of cases reported to AVN = 24** | | | |
| **Clinical characteristics** | **Adolescents** | **Adults** | **P value** |
| Number of cases; N (%) | 4 (16.7) | 20 (83.3) |  |
| Median age in years; IQR | 15; 14.5-15.2 | 47; 33.8-51.5 | **0.002** |
| Male; N (%) | 3 (75) | 11 (55) | 0.615 |
| Severity of reactions; N (%)  Grade 2  Grade 3  Grade 4 | 1 (25)  3 (75)  0 (0) | 11 (55)  9 (45)  0 (0) | 0.590 |
| Cofactors; N (%)  *Data available (N)* | 2 (66.7)  *(N=3)* | 12 (60)  *(N=20)* | 0.179 |
| **Atopic comorbidities; N (%)** | | | |
| Asthma  *Data available (N)* | 2 (66.7)  *(N=3)* | 6 (30)  *(N=20)* | 0.269 |
| Birch pollen allergy  *Data available (N)* | 2 (66.7)  *(N=3)* | 7 (38.9)  *(N=18)* | 0.553 |
| Prior allergy diagnosis to culprit allergen  *Data available (N)* | 0 (0)  *(N=3)* | 0 (0)  *(N=19)* | - |
| History of OAS  *Data available (N)* | 2 (66.7)  *(N=3)* | 4 (20)  *(N=20)* | 0.155 |

Data were complete for all cases for age, sex, and severity of reactions. Hazelnut CRD were available for a subset of cases (adolescents N=7, 63.6%; adults N=12, 30%). "Data available (N)" denotes the number of cases with non-missing data for that variable. Percentages are calculated using available data as the denominator (complete-case analysis, no imputation. AVN, Allergy-Vigilance Network; CRD, Component-resolved diagnostics; Interquartile range (25^th^ and 75^th^ percentile); OAS, Oral allergy syndrome; PR-10, Pathogenesis-related protein 10; ^a^Values as measured on ImmunoCAP, Thermo Fisher Scientific. ^b^P values are reported for descriptive completeness and consistency of table format. Comparisons are underpowered and potentially unstable and are interpreted as descriptive only.

**Supplemental Table 9 Clinical phenotypes of food-induced anaphylaxis attributed to main seeds allergen in adolescents vs. adults**

| **Sesame**  **Total number of cases reported to AVN = 46** | | | |
| --- | --- | --- | --- |
| **Clinical characteristics** | **Adolescents** | **Adults** | **P value** |
| Number of cases; N (%) | 13 (28.3) | 33 (71.7) |  |
| Median age in years; IQR | 16; 14-18 | 40; 36-50 | **<0.001** |
| Male; N (%) | 6 (46.2) | 15 (45.5) | 1.000 |
| Severity of reactions; N (%)  Grade 2  Grade 3  Grade 4 | 9 (69.2)  4 (30.8)  0 | 18 (54.5)  15 (45.5)  0 | 0.510 |
| Cofactors; N (%)  *Data available (N)* | 5 (38.5)  *(N=13)* | 11 (35.5)  *(N=31)* | 1.000 |
| **Atopic comorbidities; N (%)** | | | |
| Asthma  *Data available (N)* | 4 (36.4)  *(N=11)* | 7 (25)  *(N=28)* | 0.694 |
| Birch pollen allergy  *Data available (N)* | 3 (27.3)  *(N=11)* | 1 (3.7)  *(N=27)* | 0.065 |
| Prior allergy diagnosis to culprit allergen  *Data available (N)* | 4 (36.4)  *(N=11)* | 3 (10.7)  *(N=28)* | 0.083 |
| History of OAS  *Data available (N)* | 1 (7.7)  (N=13) | 2 (7.1)  *(N=28)* | 1.000 |
| **Pine nut**  **Total number of cases reported to AVN = 28** | | | |
| **Clinical characteristics** | **Adolescents** | **Adults** | **P value** |
| Number of cases; N (%) | 13 (46.4) | 15 (53.6) | - |
| Median age in years; IQR | 14; 13-16 | 32; 27-50.5 | **<0.001** |
| Male; N (%) | 7 (53.8) | 9 (60) | 0.841 |
| Severity of reactions; N (%)  Grade 2  Grade 3  Grade 4 | 11 (84.6)  2 (15.4)  0 | 9 (60)  6 (40)  0 | 0.221 |
| Cofactors; N (%)  *Data available (N)* | 1 (7.7)  *(N=13)* | 6 (50)  *(N=12)* | **0.030** |
| **Atopic comorbidities; N (%)** | | | |
| Asthma  *Data available (N)* | 3 (25)  *(N=12)* | 3 (23.1)  *(N=14)* | 1.000 |
| Birch pollen allergy  *Data available (N)* | 3 (25)  *(N=12)* | 0  *(N=12)* | 0.217 |
| Prior allergy diagnosis to culprit allergen  *Data available (N)* | 1 (9.1)  *(N=11)* | 1 (7.1)  *(N=14)* | 1.000 |
| History of OAS  *Data available (N)* | 1 (8.3)  *(N=12)* | 3 (21.4)  *(N=14)* | 0.598 |

Data were complete for all cases for age, sex, and severity of reactions. "Data available (N)" denotes the number of cases with non-missing data for that variable. Percentages are calculated using available data as the denominator (complete-case analysis, no imputation. AVN, Allergy-Vigilance Network; IQR, Interquartile range (25^th^ and 75^th^ percentile); OAS, Oral allergy syndrome.

**REFERENCES**

1.	World Health Organization. Plant-based diets and their impact on health, sustainability and the environment: a review of the evidence. 2021 [cited 2026 Jan 14]. Available from: https://www.who.int/europe/publications/i/item/WHO-EURO-2021-4007-43766-61591.
